# Tyrosine Phosphorylation Profiling Revealed the Signaling Network Characteristics of CAMKK2 in Gastric Adenocarcinoma

**DOI:** 10.3389/fgene.2022.854764

**Published:** 2022-05-13

**Authors:** Mohd. Altaf Najar, Mohammad Arefian, David Sidransky, Harsha Gowda, T. S. Keshava Prasad, Prashant Kumar Modi, Aditi Chatterjee

**Affiliations:** ^1^ Center for Systems Biology and Molecular Medicine, Yenepoya (Deemed to be University), Mangalore, India; ^2^ Department of Oncology and Otolaryngology-Head and Neck Surgery, Johns Hopkins University School of Medicine, Baltimore, MD, United States; ^3^ Institute of Bioinformatics, International Technology Park, Bangalore, India; ^4^ Manipal Academy of Higher Education (MAHE), Manipal, India

**Keywords:** CAMKK2, mass spectrometry, gastric cancer, PTK2, STAT3

## Abstract

Calcium/calmodulin-dependent protein kinase kinase 2 (CAMKK2) is a serine/threonine protein kinase which functions *via* the calcium-triggered signaling cascade with CAMK1, CAMK4, and AMPKα as the immediate downstream substrates. CAMKK2 is reported to be overexpressed in gastric cancer; however, its signaling mechanism is poorly understood. We carried out label-free quantitative tyrosine phosphoproteomics to investigate tyrosine-mediated molecular signaling associated with CAMKK2 in gastric cancer cells. Using a high-resolution Orbitrap Fusion Tribrid Fourier-transform mass spectrometer, we identified 350 phosphotyrosine sites mapping to 157 proteins. We observed significant alterations in 81 phosphopeptides corresponding to 63 proteins upon inhibition of CAMKK2, among which 16 peptides were hyperphosphorylated corresponding to 13 proteins and 65 peptides were hypophosphorylated corresponding to 51 proteins. We report here that the inhibition of CAMKK2 leads to changes in the phosphorylation of several tyrosine kinases such as PKP2, PTK2, EPHA1, EPHA2, PRKCD, MAPK12, among others. Pathway analyses revealed that proteins are differentially phosphorylated in response to CAMKK2 inhibition involved in focal adhesions, actin cytoskeleton, axon guidance, and signaling by VEGF. The western blot analysis upon inhibition and/or silencing of CAMKK2 revealed a decrease in phosphorylation of PTK2 at Y925, c-JUN at S73, and STAT3 at Y705, which was in concordance with the mass spectrometry data. The study indicates that inhibition of CAMKK2 has an anti-oncogenic effect in gastric cells regulating phosphorylation of STAT3 through PTK2/c-JUN in gastric cancer.

## Introduction

Tyrosine kinases (TKs) are important mediators of signaling cascades, which regulate diverse biological processes like growth, differentiation, metabolism, and apoptosis in response to external and internal stimuli (Paul and Mukhopadhyay, 2004). Tyrosine kinase signaling pathways normally prevent deregulated proliferation or contribute to sensitivity toward apoptotic stimuli (Huang et al., 2021; Moon, 2021; Saraon et al., 2021). Dysregulation of TKs is involved in different malignancies (Paul and Mukhopadhyay, 2004). Tyrosine kinases represent a major portion of all known proteins that play a transforming role in a plethora of cancers (Paul and Mukhopadhyay, 2004). The more literature we search on cellular signaling, the more we find TKs involvement in cellular signaling circuits implicated in cancer development (Lanzi and Cassinelli, 2020; Muller and Schmidt-Arras, 2020; Barbosa et al., 2021; Liu et al., 2021).

Different signaling pathways are associated with gastric cancer (GC) pathogenesis. We and others have shown the importance of MAP kinase signaling pathways in GC pathogenesis (Magnelli et al., 2020; Najar et al., 2021b). PI3K/AKT/mTOR signaling pathway dysregulation has been reported to affect gastric cancer prognosis and metastasis through epigenetic alterations, such as DNA methylation and histone modifications (Fattahi et al., 2020). Dysregulation of Rho/ROCK signaling pathway plays an important role in invasion and metastasis of GC (Matsuoka and Yashiro, 2014). Activation of p38δ/pMAPKAPK2/pHSP27 signaling pathway by G antigen 7B (GAGE7B) is involved in tumor growth and metastasis in GC (Shi et al., 2019). Moreover, activating of the PTEN/AKT/mTOR signaling pathway promotes angiogenesis and EMT in GC.

The fibroblast growth factor (FGF) family contains multiple secreted proteins that interact with signaling tyrosine kinase FGF receptors (FGFRs), resulting in its activation (Ornitz and Itoh, 2015). Activated FGFRs phosphorylate specific tyrosine residues that mediate interaction with the cytosolic adaptor proteins SOS-RAS-RAF-MEK-ERK signaling cascade (Katoh, 2007). FGF7 is a member of the FGF family that is reported to stimulate proliferation of diffuse-type gastric cancer (Nakazawa et al., 2003), and FGFR2 is reported to be overexpressed in gastric cancer. In addition, the FGF7-FGFR2 signaling cascade is reported to play a crucial role in the development and progression of diffuse-type gastric cancer (Katoh, 2003). The epidermal growth factor receptor (EGFR), another tyrosine kinase receptor, is involved in the regulation of gastric mucosa proliferation and progression of gastric carcinomas through the Ras/MEK-ERK signaling pathway, and its overexpression is associated with poor prognosis in gastric cancer (Yoshida et al., 1989; Matsubara et al., 2008). Our group has recently reported regulation of the MEK-ERK pathway by CAMKK2 in gastric cancer (Najar et al., 2021a). The above studies indicate the cross talk between the signaling cascade mediated by serine/threonine kinases (STKs) and tyrosine kinases.

CAMKK2 is a serine/threonine kinase, with well-characterized substrates such as calcium/calmodulin-dependent protein kinase type 1 (CAMKI), CAMKIV, and 5′AMP-activated protein kinase catalytic subunit alpha-2 (AMPK) (Tokumitsu et al., 1997). CAMKK2 phosphorylates CAMKIV, CAMKI, and AMPK on the activation loop of Thr residues (Thr-200, Thr-177, and Thr-172, respectively), which increases their kinase activities (Anderson et al., 1998). CAMKK2 is reported to activate a number of kinases through CAMKK2-AMPK and CAMKK2-CAMKIV pathways involved in different cellular functions (Racioppi and Means, 2012). Apart from the role of CAMKK2 in normal pathophysiological conditions, it is reported to be overexpressed in multiple cancers, such as gastric cancer (Fu et al., 2015; Lin et al., 2015; Subbannayya et al., 2015; Racioppi et al., 2019). Our group has shown in detail the regulation of the molecular profile associated with CAMKK2 (Najar et al., 2021b) and signaling pathways regulated by CAMKK2 in gastric cancer (Najar et al., 2021a). Studies have indicated that signaling cascades involve cross talk between STKs and TKs (Marin et al., 1999; Katoh, 2007; Pinto et al., 2018; Sahu et al., 2018). Since several studies have pointed out a cross talk between STKs and TKs, we sought to study how CAMKK2 regulates tyrosine phosphorylation and the associated signaling cascades in gastric cancer.

In this study, we determined the regulatory roles of CAMKK2 through tyrosine phosphorylation in gastric adenocarcinoma (AGS) cells. We carried out a label-free quantitative phosphotyrosine proteomic analysis to investigate the role of phosphotyrosine signaling in CAMKK2-mediated progression of GC in gastric cancer (AGS) cells.

## Materials and Methods

### Reagents

The PTMScan® Phospho-Tyrosine Rabbit mAb (P-Tyr-1000) Kit, AMPK, phospho-AMPK at T172, CAMKK2, PTK2, phospho-PTK2 at Y925, phospho STAT3 at Y705, C-Jun, phospho C-Jun at T73 antibodies were obtained from Cell Signaling Technology (Danvers, United States). Anti-β actin–HRP conjugated antibody was purchased from Sigma-Aldrich, United States; STO-609 (7-oxo-7H-benzimidazo [2,1-a]benz[de] isoquinoline-3-carboxylic acid–acetic acid), a CAMKK2 inhibitor, was purchased from Santa Cruz Biotechnology, Inc. (Texas, United States). TPCK-treated trypsin was obtained from Worthington Biochemical Corp. (Lakewood, NJ). Dulbecco’s Modified Eagle Medium (DMEM) with high glucose, fetal bovine serum (FBS), and antibiotic–antimitotic solution were purchased from Gibco (Thermo Fisher Scientific, United States). All other consumables used in the study were from Sigma Aldrich, United States.

### MTT Cell Proliferation Assay

To determine the effect of STO-609 on AGS cells, the MTT (3-(4,5-dimethyl thiazolyl-2)-2,5-diphenyltetrazolium bromide) assay was carried out as described previously (Xiao et al., 2014). Briefly, the cells were seeded at a density of 8 × 10^3^ and treated with STO-609 at varying concentrations (0, 5, 15, 25, and 50 µM) for 48 h. After incubation, MTT reagent was added and incubated for 2–4 h until a purple precipitate was formed. The purple crystals were solubilized using 100 µl of a solubilizing reagent (DMSO:ethanol 1:1) and kept at room temperature for 2 h. Furthermore, the absorbance was read at 570 and 650 nm.

### Cell Culture

AGS cells were obtained from the American Type Culture Collection (ATCC, Manassas, United States). AGS cells were cultured in the DMEM containing 10% FBS and 1% antibiotic–antimycotic mixture and maintained in a humidified incubator at 37°C with 5% CO_2_. When the cells reached 70% confluence, the cells were subjected to serum starvation for 8 h. Post–serum starvation, the cells were treated with DMSO as the control and 18.5 μM STO-609 as CAMKK2 inhibited in regular DMEM for 2 h. Following 2 h of treatment, the cells from both the conditions were washed with ice-cold 1× phosphate buffer saline thrice and harvested in lysis buffer.

### Colony Formation Assays

For colony formation, AGS cells were seeded at a density of 3 × 10^3^ cells into a six-well plate with complete media. After 24 h, the cells were treated with 18.5 μM STO-609, while the cells treated with dimethyl sulfoxide (DMSO) served as the control. Cell colonies were allowed to grow for 11 days. Finally, the colonies were fixed using methanol and stained with 0.3% crystal violet. The number of colonies per well were counted. All experiments were done in three biological and three technical replicates.

### Invasion Assay

The effect of CAMKK2 inhibition/silencing on the invasion potential of AGS cells was assessed *in vitro* in a transwell system (BD Biosciences, San Jose, CA) using Matrigel-coated filters. 18.5 μM STO-609 or CAMKK2 siRNA was used to inhibit/silence CAMKK2, and DMSO/scrambled siRNA was used as the control. The cells at a density of 2 × 10^4^ were suspended in 500 µl of serum-free media and seeded on the Matrigel-coated PET membrane in the upper compartment. The lower compartment was filled with complete growth media, and the plates were incubated at 37°C for 48 h. At the end of incubation, the upper surface of the membrane was wiped with a cotton-tip applicator to remove nonmigratory cells. The cells that migrated to the bottom side of the membrane were fixed and stained using 4% methylene blue. Each measurement was performed in duplicate. All experiments were repeated thrice.

### Migration Assay

The *in vitro* scratch wound healing assay was performed in six-well cell culture plates. The cells were seeded in six-well plates and grown until 70% confluence. Next, a pipette tip was used to scratch (wound) the monolayer, and the remaining cells were washed with a serum-free medium. The cells were treated with 18.5 μM STO-609, and DMSO was taken as the control. The cells were allowed to grow until the closure of the scratch (48 h); images were taken at different time points by a Primovert inverted microscope (Carl Zeiss, Germany).

### Cell Proliferation Assay

The cell proliferation assay was performed by using crystal violet staining method. A density of 5 × 10^4^ AGS cells were seeded for each well in six-well plates, the cells were treated with 18.5 μM STO-609, and DMSO was taken as the control. We also knockdown CAMKK2 by using CAMKK2 siRNA and studied the effect of CAMKK2 knockdown on cellular proliferation. The cells were transfected with CAMKK2 siRNA and scrambled siRNA (transfection described under siRNA transfection). They were allowed to grow for 72 h and were washed with PBS, fixed with methanol, and stained with 3% crystal violet. The live cells were stained with crystal violet, while dead cells were washed away as they did not remain adherent. Images were taken with a Primovert inverted microscope (Carl Zeiss, Germany). Each measurement was performed in duplicate, and the experiments were repeated thrice.

### siRNA Transfection

siRNA transfection was carried out using ON-TARGETplus SMARTpool control siRNA and CAMKK2 siRNA (Dharmacon, Lafayette, United States). AGS cells were transfected as previously described by using RNAiMAX (Invitrogen, Grand Island, NY) (Najar et al., 2021b). They were plated at a density of 60,000 cells/well in six-well plates followed by transfection with 20 nM of CAMKK2 siRNA and control siRNA in Opti-MEM media (Gibco, CA, United States) using RNAiMAX. The cells were transfected with CAMKK2 siRNA and control siRNA in Opti-MEM media using Lipofectamine RNAiMAX transfection reagent. After transfection, the media was changed to complete media (DMEM +10% FBS) and the cells were allowed to grow for 48 h. Finally, the cells were harvested using lysis buffer, and lysates were used for immunoblotting experiments to check protein expression.

### Western Blot Analysis

AGS cells were cultured in DMEM containing 10% FBS and antibiotic–antimycotic solution at 37 °C with 5% CO_2_ in a humidified incubator. The cells were treated with a vehicle control (DMSO) and 18.5 μM STO-609 for 1 h. They were lysed by sonication in a lysis buffer (50 mM TEABC, pH 8.0, 2% SDS, 1 mM sodium orthovanadate, 2.5 mM sodium pyrophosphate, 1 mM β-glycerophosphate) followed by centrifugation. The protein lysates were resolved using SDS-PAGE, and the western blot analysis was performed using total AMPK, phospho-AMPK, CAMKK2, total PTK2, phospho-PTK2, total c-JUN, phospho-c-JUN, and phospho STAT3 antibodies. β-actin was used as a loading control.

### Cell Lysis and Protein Digestion

DMSO (vehicle control) and STO-609–treated AGS cells were lysed in lysis buffer (8 M urea, 100 mM HEPES buffer with protease and phosphatase inhibitors cocktail), sonicated, and centrifuged at 16,000×*g* for 20 min. Protein concentration was determined using the BCA assay (Pierce, Waltham, MA). Equal amounts of protein (6 mg) were taken and processed for reduction and alkylation with 10 mM DTT for 20 min at 60°C and 20 mM iodoacetamide for 20 min at room temperature, respectively. The samples were diluted to decrease the urea concentration to 1 M. Proteins were digested by TPCK-treated trypsin in the enzyme to protein ratio of 1:20 for 16 h at 37°C. The reaction was quenched with the addition of 0.1% trifluoroacetic acid (TFA). The digested peptides were cleaned by C18 StageTips and vacuum dried before immunoaffinity purification of tyrosine phosphopeptides.

### Immunoaffinity Purification of Tyrosine Phosphopeptides

We used the PTMScan® Phospho-Tyrosine Rabbit mAb (P-Tyr-1000) Kit (Catalog No. 8803, Cell Signaling Technology, United States) for immunoaffinity purification of tyrosine phosphopeptides. Immunoaffinity purification of tyrosine phosphopeptides was carried out as per the manufacturer’s protocol. Briefly, lyophilized peptides were dissolved in IAP buffer containing 50 mM MOPS, pH 7.2, 10 mM sodium phosphate, and 50 mM NaCl. Before phosphotyrosine enrichment, the P-Tyr-1000 beads were washed twice with IAP buffer at 4°C. The peptide mixture was then incubated with P-Tyr-1000 beads for 30 min with gentle rotation. To remove nonspecifically bound peptides, the beads were washed thrice with ice-cold IAP buffer and twice with ice-cold water. The elution of enriched peptides from the beads was carried out at room temperature using 0.15% TFA. This step was repeated twice. This was followed by the cleanup of the samples using C18 StageTips.

### Liquid Chromatography with Tandem Mass Spectrometry Analysis of Enriched Peptides

Enriched phosphotyrosine peptides were analyzed on Orbitrap Fusion Tribrid mass spectrometer (Thermo Fisher Scientific, Bremen, Germany) interfaced with Easy-nLC 1200 nanoflow liquid chromatography system (Thermo Fisher Scientific, Odense, Denmark). Peptide digests were reconstituted in 0.1% formic acid and loaded onto the trap column (75 μm × 2 cm) at a flow rate of 3 μl/min. The peptides were separated on an analytical column (75 μm × 50 cm) at a flow rate of 300 nL/min using a step gradient of 5–40% solvent B (0.1% formic acid in 80% acetonitrile) for the first 98 min and 40–60% for the next 7 min minutes, 60–100% for 15 min—the total run time was set to 120 min. The mass spectrometer was operated in the data-dependent acquisition mode. A survey full scan MS (from m/z 350–1700) was acquired in the Orbitrap with a resolution of 120,000 at 400 m/z. The data were acquired in top speed at charge state ≥2, and isolated and fragmented using stepped collision energy with ±5 fragmentation with 34% normalized collision energy and detected at a mass resolution of 30,000 at 400 m/z. Dynamic exclusion was set for 30 s with a 10-ppm mass window.

### Data Analysis

The MS/MS searches were carried out using both MASCOT and SEQUEST search algorithms against RefSeq human protein database (version 94 with common contaminants) using Proteome Discoverer (Version 2.2.0.288, Thermo Fisher Scientific, Bremen, Germany). The workflow for both algorithms included spectrum selector, MASCOT, SEQUEST search nodes, peptide validator, event detector, precursor quantifier, and ptmRS nodes. Oxidation of methionine, phosphorylation at serine, threonine, and tyrosine (+79.966 Da), and carbamidomethylation of cysteine were set as a fixed modification. MS and MS/MS mass tolerances were set to 10 ppm and 0.05 Da, respectively. Trypsin was specified as protease and a maximum of two missed cleavages were allowed. Target-decoy database searches were used to calculate the false discovery rate (FDR), and for peptide identification, the FDR was set at 1%. Feature mapper and precursor ion quantifier were used for label-free quantification. The ptmRS node in the Proteome Discoverer was used for the probability and confidence of the phosphorylation site. The normalization was done based on the total peptide amount in the quantification node of the Proteome Discoverer. Phosphopeptides with >95% localization probability were considered for further analysis.

### Bioinformatics Analysis

The differentially phosphorylated proteins upon inhibition of CAMKK2 were used for gene ontology analysis using the DAVID bioinformatics functional annotation tool (Huang et al., 2009). The pathway analysis on significantly altered proteins was done using the Reactome database. The domain analysis was done for the hypophosphorylated sites upon inhibition of CAMKK2 using MotifFinder, an online tool against the NCBI-CDD conserved domain database (https://www.genome.jp/tools/motif/MOTIF.html) with an E-value cutoff of 1.0. The SankeyMATIC (BETA) (http://sankeymatic.com/build/) was used to represent the pathway data. Rstudio with ggplot2 was used to make bubble plot, volcano plot, violin plot, and generation of heat maps. For kinases KinMap (http://www.kinhub.org/kinmap/), an online tool, was used for the visualization of the kinase tree.

### Availability of Mass Spectrometry Data

The Proteome data were deposited to the ProteomeXchange Consortium (http://proteomecentral. proteomexchange.org) *via* the PRIDE partner repository with the data set identifier PXD032001.

## Results

**FIGURE 1:**
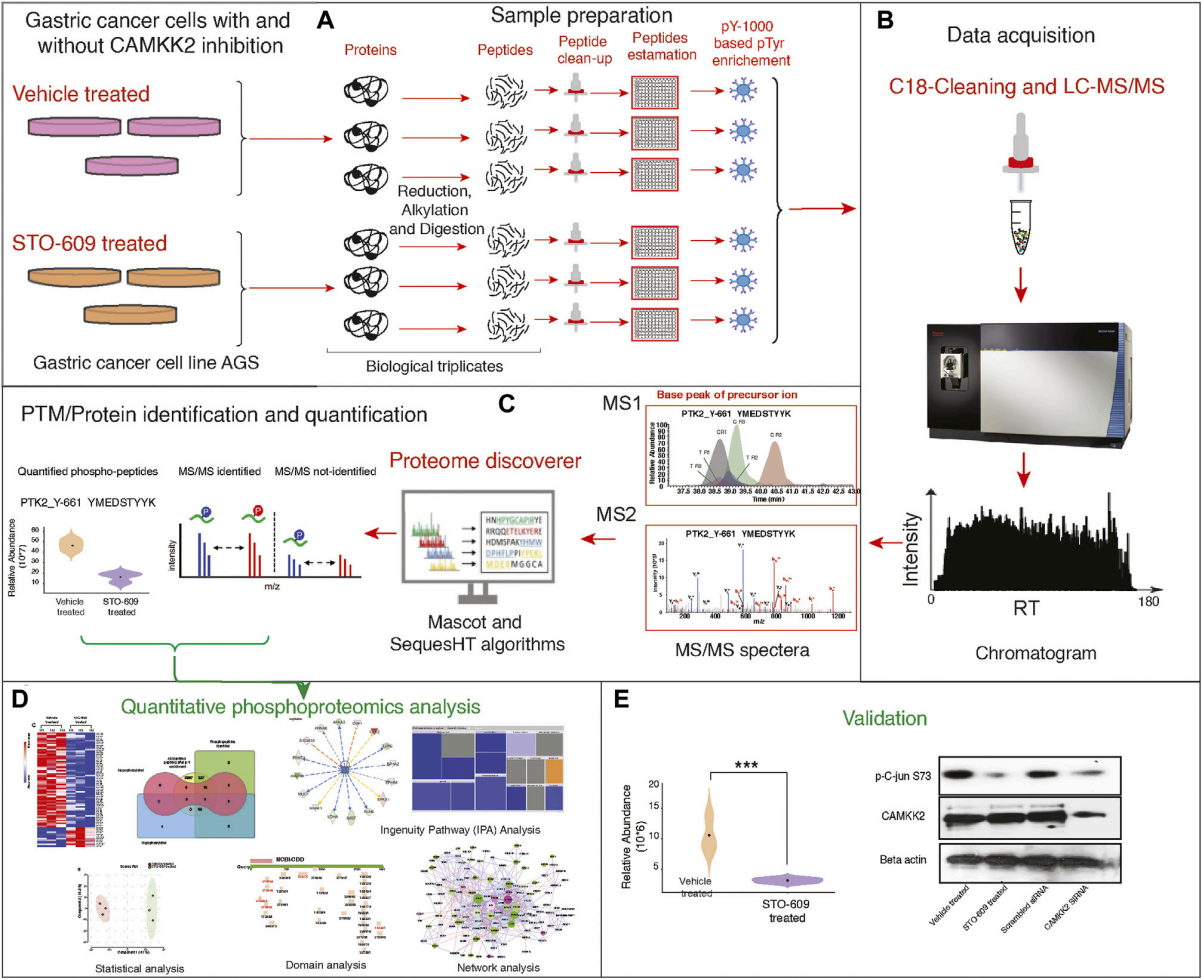
Detailed experimental workflow for phospho-(Y)-proteomic analysis, data analysis, and functional validation in gastric cancer cells between STO-609-treated and DMSO control conditions. **(A)** Schematic representation adapted for sample preparation for identification and quantification of phospho-(Y)-proteomic changes in gastric cancer cells (AGS) treated with STO-609 and vehicle control (DMSO). **(B)** Schematic representation LC-MS/MS analysis. **(C)** Schematic representation for PTM/protein identification and quantification from LC-MS/MS data using proteomic platform Proteome Discoverer. **(D)** Bioinformatics analysis/data analysis of proteomic data obtained in gastric cancer cells (AGS) treated with STO-609 and vehicle control (DMSO). **(E)** Validation of protein expression identified from proteomic data analysis.

Our group has reported overexpression of CAMKK2 in gastric cancer (Subbannayya et al., 2015). Our previous studies have reported that CAMKK2 regulates multiple pathways involved in cellular transformation in gastric cancer cells (Najar et al., 2021b) and activates CDK by CAMKK2 through the MEK-ERK pathway, promoting cellular proliferation in gastric cancer cells (Najar et al., 2021a). Several studies in cancer have reported activation of the MEK-ERK pathway by TKs (Fang and Richardson, 2005; Dhillon et al., 2007). We aimed to study tyrosine kinase signaling cascade in gastric cancer by CAMKK2 by analyzing the phosphotyrosine protein profiling in gastric cancer cells *via* inhibition of CAMKK2. AGS cells were treated with STO-609, a CAMKK2 inhibitor, followed by phosphotyrosine proteomic analysis and functional validation as shown in Figure 1.

### Quantitative Tyrosine Phosphoproteomic Analysis Upon Inhibition of CAMKK2

**FIGURE 2:**
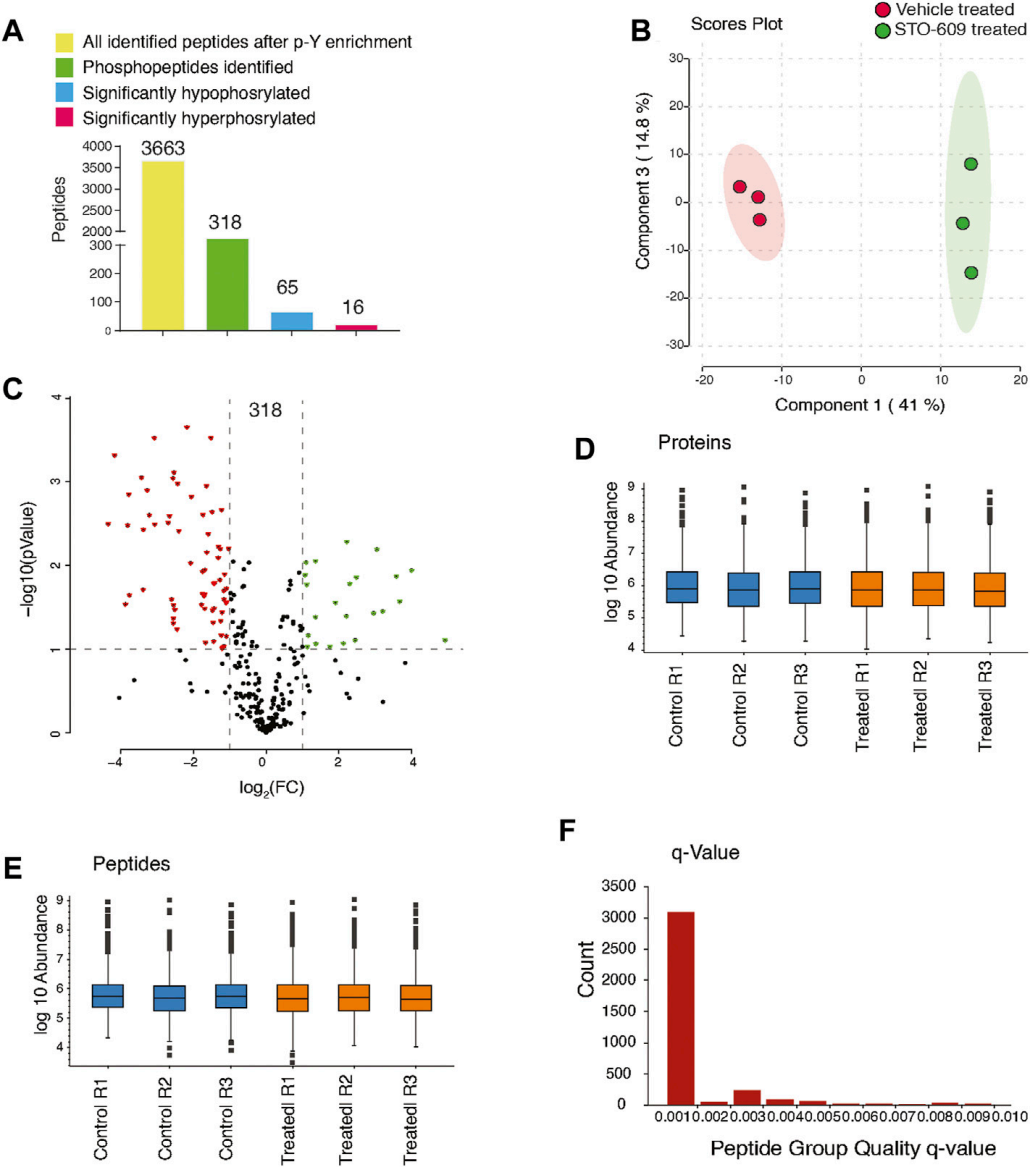
Inhibition of CAMKK2 results in change in tyrosine phosphoproteome in gastric cancer cells. **(A)** Bar graph summarizes phospho-Y-proteomic changes in gastric cancer cells (AGS) treated with 18.5 μM STO-609 and vehicle control. **(B)** Principal component analysis of control and CAMKK2-inhibited AGS cells. **(C)** Volcano plot showing the distribution of phosphopeptides upon STO-609 treatment 18.5 µM for 2 h. **(D)** Box plot showing the sum of protein abundances between the control and STO-609–treated AGS cells across the triplicates. **(E)** Box plot showing the sum of peptides abundances between the control and STO-609–treated AGS cells across the triplicates. **(F)** The bar graph shows the q-value of peptides.

To characterize CAMKK2-mediated tyrosine signaling mechanism in gastric cancer cells, we used label-free–based quantitative phosphoproteomic analysis (LFQ) as shown in Figure 1A. The LC-MS/MS analysis resulted in the identification of 13,187 peptide-spectral matches with a false discovery rate (FDR) of 1%. PhosphoRS probability cutoff of 95% was used for unambiguous localization of phosphorylation sites, which identified 318 unique phosphopeptides corresponding to 350 phosphorylation sites mapping to 157 proteins (Supplementary Table S1). Using a 1.5-fold cutoff for hyperphosphorylation and the 0.67-fold cutoff for decreased phosphorylation (hypophosphorylation) with a *p*-value of ≤0.05, we identified 16 hyperphosphorylated tyrosine phosphopeptides corresponding to 13 proteins and 65 hypophosphorylated tyrosine phosphopeptides correspond to 51 proteins upon inhibition of CAMKK2 (Supplementary Table S2, Figure 2A). Data were acquired in biological triplicates, which showed a good correlation as evidenced by the principal component analysis (Figure 2B) and reproducibility as shown in the Venn diagram (Supplementary Figure S6). A volcano plot showing the distribution of significantly dysregulated tyrosine phosphoproteins upon inhibition of CAMKK2 in gastric cancer is depicted in Figure 2C. Label-free quantification showed the sum of protein and peptide abundances to be the same between vehicle-treated and STO-609–treated samples (Figures 2D,E). Q-values are also known as the minimum false discovery rate (FDR), a parameter used for accurate identification in LC-MS/MS data. A less q-value represents more confident data in terms of true identification with minimum false discovery. Most of the peptides identified in our study showed a q-value less than 0.001% (Figure 2F), indicating high confidence data.

### Functional Analysis of CAMKK2-Regulated Tyrosine Phosphoproteome

**FIGURE 3:**
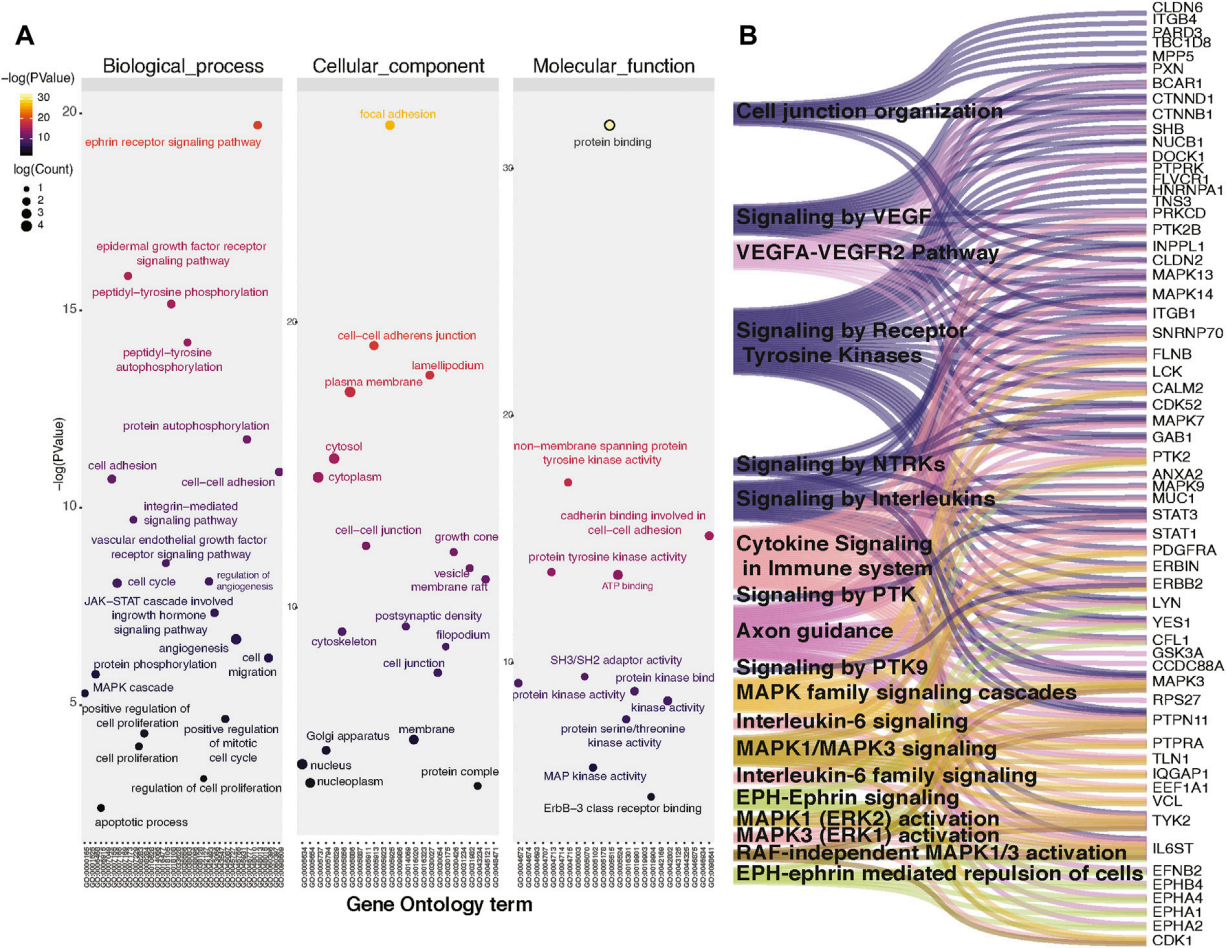
Inhibition of CAMKK2 regulates tyrosine phosphoproteome involved in different cellular processes and pathways. **(A)** Gene ontology enrichment of biological process, cellular component, and molecular function associated with hypophosphorylated proteins in gastric cancer cells (AGS) upon CAMKK2 inhibition. **(B)** Pathway analysis associated with hypophosphorylated proteins in AGS cells upon CAMKK2 inhibition.

**FIGURE 4:**
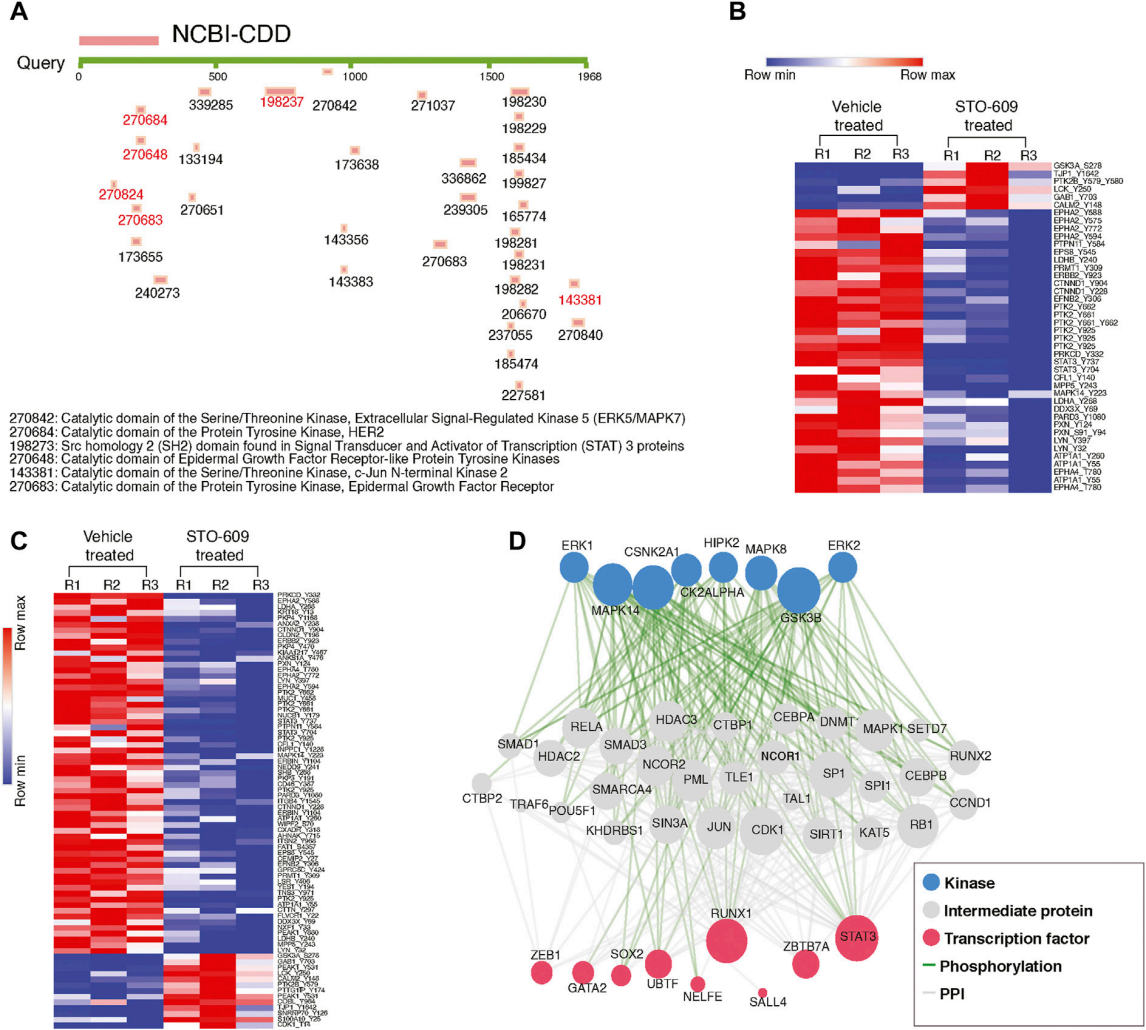
Inhibition of CAMKK2 decreases the phosphorylation of tyrosine kinases in gastric cancer cells. **(A)** Domain analysis of hypophosphorylated peptides using MotifFinder tool against Conserved Domains Database (CDD). **(B)** Heat map showing the significantly altered phospho-Y-peptides of different kinases in gastric cancer cells (AGS) upon CAMKK2 inhibition. **(C)** Heat map showing the significantly altered phospho-Y-peptides of different non-kinases proteins in gastric cancer cells (AGS) upon CAMKK2 inhibition. **(D)** eXpression2Kinases analysis of hypo-Y-phosphorylated proteins on CAMKK2 in gastric cancer. Network shows kinases, protein–protein interaction, and transcriptional factors enriched in hypo-Y-phosphorylated data set in gastric cancer cells treated with STO-609.

Since we observed widespread alterations of tyrosine phosphorylation upon inhibition of CAMKK2, we performed bioinformatics analysis for the differentially phosphorylated proteins to categorize them based on their biological process, cellular localization, and biological function. This analysis revealed that most of the hypophosphorylated proteins upon inhibition of CAMKK2 are involved in the ephrin receptor signaling pathway, epidermal growth factor receptor signaling pathway, peptidyl-tyrosine autophosphorylation, and protein autophosphorylation (Figure 3A, biological process). The cellular component analysis revealed most of the hypophosphorylated proteins were localized in the cytoplasm, followed by focal adhesion, plasma membrane, nucleus, cytoskeleton, and growth cone (Figure 3A, cellular components). Molecular function analysis of hypophosphorylated proteins shows that they are involved in protein binding, non-membrane spanning protein tyrosine kinase activity, and ATP binding (Figure 3A, molecular functions). Furthermore, pathway analysis using the Reactome database to group hypophosphorylated proteins into different canonical pathways led to the identification of several signaling pathways regulated by tyrosine kinase. Some of the top pathways identified are the VEGFA–VEGFR2 pathway, MAPK signaling cascade, and cytokine signaling in the immune system (Figure 3B). We have further performed interactome analysis using the STRING database and Cytoscape tool. Our analysis showed a high interaction between differentially phosphorylated proteins upon inhibition of CAMKK2 (Supplementary Figure S1A). The domain analysis for peptide sequences that were hypophosphorylatedat the tyrosine residue upon inhibition of CAMKK2 using the MotifFinder tool resulted in identifying 32 domains, most of which have a catalytic function. We identified catalytic domains such as serine/threonine kinase, extracellular signal-regulated kinase 5 (ERK5/MAPK7), protein tyrosine kinase HER2, and Src homology 2 (SH2). Some of the domains identified are involved in STAT3 activation and c-Jun N-terminal kinase 2 activation (Figure 4A). Our data showed that CAMKK2 regulates tyrosine phosphorylation of proteins involved in different cellular signaling.

### Inhibition of CAMKK2 Results in Decreased Phosphorylation of Tyrosine Kinases in Gastric Cancer

We studied the phosphorylation status of kinases upon inhibition of CAMKK2. In our phosphotyrosine data, we observed a significant decrease in phosphorylation of focal adhesion kinase 1 (PTK2) at Y662, ephrin type-A receptor 1 precursor (EPHA1) at Y781, ephrin type-A receptor 2 (EPHA2) at Y588, ephrin type-A receptor 4 (EPHA4) at T780, ephrin type-B receptor 4 (EPHB4) at Y793, Receptor tyrosine–protein kinase erbB-2 (ERBB2) at Y923, mitogen-activated protein kinase 14 (MAPK14) at Y223, and mitogen-activated protein kinase 7 (MAPK7) at T219, Y221 (Figure 4B and Table 1). In addition to kinases, we also observed a decrease in the phosphorylation of several non-kinase proteins at tyrosine residue upon inhibition of CAMKK2 in gastric cancer. These include plakophilin-4 (PKP4) at Y415, Y470, and Y1168; plakophilin-2 (PKP2) at S164; plakophilin-3 (PKP3) at Y191, annexin A2 (ANXA2) at Y238, nucleobindin-1 (NUCB1) at Y179, cofilin-1 (CFL1) at Y140, enhancer of filamentation 1 (NEDD9) at Y241 and Y345, Erbin (ERBIN) at Y1104, protein arginine N-methyltransferase 1 (PRMT1) at Y309, and ATP-dependent RNA helicase DDX3X (DDX3X) at Y69 (Figure 4C). These proteins are reported to play an essential role in multiple cancers.

**TABLE 1:** List of significantly altered tyrosine‐phosphorylation of kinases upon inhibition of CAMKK2 in gastric cancer, which were not reported earlier to be regulated by CAMKK2.

Gene	Sequence	P-site	PSM^a^	FC (T/C)	*p*-Value
EPHA1	[R].LLDDFDGTYETQGGK.[I]	Y781	7	0.3664	0.0403
EPHA2	[K].SEQLKPLKTYVDPHTYEDPNQAVLK.[F]	Y588	56	0.4690	0.0094
EPHA4	[R].VLEDDPEAAYTTR.[G]	T780	87	0.5328	0.0045
EPHB4	[R].FLEENSSDPTYTSSLGGK.[I]	Y793	6	0.3299	0.0021
PTK2	[R].YMEDSTYYK.[A]	Y662	92	0.2202	0.0001
ERBB2	[R].LLDIDETEYHADGGKVPIK.[W]	Y923	46	0.2362	0.0047
LYN	[R].VIEDNEYTAR.[E]	Y397	46	0.4448	0.0100
MERTK	[RK].KIYSGDYYR.[Q]	Y749	15	0.7228	0.1696
MAPK14	[R].HTDDEMTGYVATR.[W]	Y223	88	0.6506	0.0088
PDGFRA	[R].SLYDRPASYK.[K]	Y787; Y793	2	0.0000	0.0583
PRKCD	[R].RSDSASSEPVGIYQGFEK.[K]	Y332	2	0.0000	0.0000
HIPK3	[K].TVCSTYLQSR.[Y]	Y359	32	0.6680	0.0306
TYK2	[R].LLAQAEGEPCYIR.[D]	Y292	4	0.6432	0.1880
YES1	[R].LIEDNEYTAR.[EQ]	Y452	78	0.7189	0.0995
MAPK7	[R].GLCTSPAEHQYFMTEYVATR.[W]	T219; Y221	9	0.0682	0.0146
PRPF4B	[K].LCDFGSASHVADNDITPYLVSR.[F]	Y849	790	0.6420	0.2628
PEAK1	[K].VPIVINPNAYDNLAIYK.[S]	Y635	15	0.2418	0.1572

a PSM = Peptide-Spectrum Match

We used the DisGeNET database in the Metascape tool and observed that the hypophosphorylated proteins upon inhibition of CAMKK2 were involved in several other malignancies. These malignancies include mesothelioma, invasive carcinoma of the breast, malignant glioma, pancreatic intraepithelial neoplasia, adenocarcinoma of the pancreas, gastrointestinal stromal tumors, uterine cancer, intestinal neoplasms, metastatic melanoma, and lymphoid leukemia (Supplementary Figure S1B,C). In addition, the Ingenuity Pathway analysis revealed that most of the hypophosphorylated proteins are involved in cancer, cellular movement, cellular growth, and tumor formation and that the hyperphosphorylated proteins are involved in cell death and survival (Supplementary Figure S2A).

### Identification of Novel Kinases Regulated by CAMKK2 in Gastric Cancer Cells

CAMKK2 is reported to be involved in the activation/phosphorylation of multiple kinases and non-kinase proteins, which are vital for cellular functions, especially cellular transformation (Najar et al., 2021c). In our phosphotyrosine proteomic data, we identified decreased phosphorylation of several kinases and non-kinase proteins at the p-Y residue upon inhibition of CAMKK2, whose regulation *via* CAMKK2 is not reported previously. These kinases play contrasting roles in cell death and cell survival by functioning as a pro-apoptotic protein during DNA damage-induced apoptosis but acting as an anti-apoptotic protein during cytokine receptor-initiated cell death. They play a tumor suppressor role by controlling cell cycle progression both at G1/S and G2/M phases in multiple cancers. One such protein is PRKCD, a calcium-independent and phospholipid and diacylglycerol (DAG)–dependent serine/threonine–protein kinase. It is overexpressed in multiple myeloma cells and ovarian carcinomas and plays an important role in plasma cell survival and ovarian carcinomas (Ni et al., 2003; Weichert et al., 2003). Our data revealed a significant decrease in phosphorylation of PRKCD at Y334 upon inhibition of CAMKK2 in gastric cancer cells.

Apart from PRKCD, our data also revealed a two-fold decrease in the phosphorylation of TYK2 at Y292 and STAT3 at Y705 and Y737 upon inhibition of CAMKK2. TYK2 is reported to be overexpressed in the prostate (Ide et al., 2008; Santos et al., 2015), ovarian (Silver et al., 2004), cervical (Zhu et al., 2009), and breast (Song et al., 2008) cancers. Reports indicate that hyperactivation of TYK2 results in the activation of STAT1/3/5 (Sanda et al., 2013). It is also reported that TYK2 phosphorylates STAT3 at Y705 and Y640 residues (Mori et al., 2017). PTK2 is also reported to activate STAT3 and is overexpressed in multiple cancers such as gastric cancer (Park et al., 2010; Francalanci et al., 2020). We have reported decreased phosphorylation of TYK2 at Y292, PTK2 at Y662, and STAT3 at Y705, Y737 upon inhibition of CAMKK2 in gastric cancer cells. We showed high interaction between these proteins using the STRING, BioGrid, and OmniPath databases in the Metascape tool. The GO analysis showed that these proteins are involved in tyrosine phosphorylation, cellular growth, and the STAT3 signaling pathway (Supplementary Figure S3A). Using the DisGeNET database, we observed that these proteins are involved in meningioma, tumor initiation, ductal carcinoma, and so on (Supplementary Figure S3B). Our results showed that CAMKK2 inhibition decreases the tumorigenesis in gastric cancer cells by reducing the phosphorylation of kinases involved in GC tumorigenesis.

### Inhibition of CAMKK2 Affects Phosphorylation of Proteins Involved in Cell Migration

Our previous studies show that inhibition of CAMKK2 decreases cellular migration of gastric cancer cells (Najar et al., 2021b). We identified hypophosphorylation of several proteins involved in cell proliferation and cell migration upon inhibition of CAMKK2 (Najar et al., 2021a). It has been reported that EPHA2 is involved in cell proliferation and migration of endothelial cells (Wiedemann et al., 2017). EPHA2 has multiple tyrosine phosphorylated sites Y772, Y575, Y588, and Y59 (Balasubramaniam et al., 2011) and phosphorylation at its activation loop Y772 increases its activity ten folds (Balasubramaniam et al., 2011). Our phosphotyrosine analysis revealed a 3.5-fold decrease in phosphorylation of EPHA2 at Y772, 3-fold decrease in phosphorylation at Y588, a 2.4-fold reduction in phosphorylation at Y575, and a 4.5-fold decrease in phosphorylation at Y594 upon inhibition of CAMKK2. EFNB2 is another essential protein that is overexpressed in pancreatic ductal adenocarcinoma. Its overexpression is involved in cell migration (Zhu et al., 2020). EFNB2 overexpression is reported in gastric cancer (Kataoka et al., 2002) and its tyrosine phosphorylation promotes invasion of gastric cancer (Tanaka et al., 2007). Our data show a decreased phosphorylation of EFNB2 at Y306 in gastric cancer cells upon inhibition of CAMKK2. In addition, we identified multiple proteins involved in cell migration that were hypophosphorylated upon inhibition of CAMKK2 (Table 2). Thus, our data suggest that decreased cell migration upon inhibition CAMKK2 may be due to decreased phosphorylation of these proteins; however, it needs further validation to prove, which is beyond the scope of the current study.

**TABLE 2:** List of significantly hypo‐phosphorylated proteins at tyrosine residue upon inhibition of CAMKK2 involved in cell migration.

Gene	Sequence	P-site	PSM^a^	FC (T/C)	*p*-Value
CLDN2	[R].SNYYDAYQAQPLATR.[S]	Y198	80	0.11	0.001
EFNB2	[R].TADSVFCPHYEK.[V]	Y306	21	0.16	0.002
CFL1	[K].HELQANCYEEVKDR.[C]	Y140	6	0.09	0.019
ERBB2	[R].LLDIDETEYHADGGKVPIK.[W]	Y923	46	0.2	0.009
LYN	[R].VIEDNEYTAR.[E]	Y397	46	0.45	0.020
EPHA4	[R].VLEDDPEAAYTTR.[G]	T780	87	0.54	0.009
YES1	[K].GAYSLSIR.[D]	Y194	20	0.37	0.032
PTPN11	[R].VYENVGLMQQQK.[S]	Y584	9	0.0000	0.043
MAPK14	[R].HTDDEMTGYVATR.[W]	Y223	88	0.66	0.017
PTK2	[R].YMEDSTYYK.[A]	Y662	92	0.23	0.000

a PSM = Peptide-Spectrum Match

### Inhibition of CAMKK2 Regulates Vascular Endothelial Growth Factor Signaling in Gastric Cancer Cells

The pathway analysis using Reactome database of hypophosphorylated proteins identified VEGF and VEGFA–VEGFR2 signaling among one of the significantly enriched pathways (Figure 3B) (Table 3). Hypophosphorylated proteins identified upon inhibition of CAMKK2 include protein kinase C delta (PRKCD), Paxillin (PXN), Catenin delta-1 (CTNND1), mitogen-activated protein kinase 14 (MAPK14), PTK2, nucleobindin-1 (NUCB1), filamin-B (FLNB), Dedicator of cytokinesis protein 1 (DOCK1), and breast cancer antiestrogen resistance protein 1 (BCAR1). The list of proteins regulated by CAMKK2 involved in VEGF and VEGFA–VEGFR2 pathways are listed in Table 4. The VEGF pathway regulates proliferation, migration, and vascular permeability of the endothelial cells (ECs). VEGF-A, an essential member of the VEGF pathway, is widely expressed by nearly all malignant tumors and is the most critical tumor angiogenesis factor (Folkman, 1971; Ferrara et al., 2003). Our results showed that inhibition might decrease migration of gastric cancer cells through the VEGF pathway.

**TABLE 3:** List of pathways significantly enriched from hypo‐phosphorylated proteins upon inhibition of CAMKK2 in gastric cancer cells.

Pathway identifier	Pathway name	#Entities found	Entities p-Value	Entities FDR
R-HSA-9006934	Signaling by receptor tyrosine kinases	35	8.7597E-14	8.584E-11
R-HSA-1059683	Interleukin-6 signaling	8	1.7371E-10	8.512E-08
R-HSA-422475	Axon guidance	29	3.6484E-09	8.939E-07
R-HSA-449147	Signaling by interleukins	30	6.7668E-09	1.326E-06
R-HSA-194138	Signaling by VEGF	14	9.7167E-09	1.584E-06
R-HSA-1280215	Cytokine signaling in immune system	44	1.164E-08	1.63E-06
R-HSA-6783589	Interleukin-6 family signaling	8	1.4004E-08	1.708E-06
R-HSA-4420097	VEGFA–VEGFR2 pathway	13	3.5198E-08	3.449E-06
R-HSA-446728	Cell junction organization	11	1.1816E-07	1.052E-05
R-HSA-3928665	EPH-ephrin–mediated repulsion of cells	8	1.3293E-06	8.652E-05
R-HSA-110056	MAPK3 (ERK1) activation	5	1.331E-06	8.652E-05
R-HSA-2682334	EPH-Ephrin signaling	10	1.9793E-06	0.0001185
R-HSA-112409	RAF-independent MAPK1/3 activation	6	3.2771E-06	0.000177
R-HSA-5684996	MAPK1/MAPK3 signaling	16	5.2327E-06	0.0002564
R-HSA-5683057	MAPK family signaling cascades	17	9.3806E-06	0.0004127
R-HSA-112411	MAPK1 (ERK2) activation	4	2.7673E-05	0.0007472
R-HSA-166520	Signaling by NTRKs	9	4.9175E-05	0.0011539
R-HSA-8848021	Signaling by PTK6	7	7.1858E-05	0.001509

**TABLE 4:** List of significantly hypo‐phosphorylated (tyrosine) proteins upon inhibition of CAMKK2 involved in VEGF and VEGFA–VEGFR pathways.

Gene	Sequence	Site	PSM^a^	FC(T/C)	P-value
PRKCD	[R].RSDSASSEPVGIYQGFEK.[K]	Y332	2	0.0001	9E-06
PXN	[R].VGEEEHVYSFPNK.[Q]	Y124	230	0.54	2E-02
CTNND1	[K].SLDNNYSTPNER.[G]	Y904	20	0.17	9E-04
SHB	[K].LPQDDDRPADEYDQPWEWNR.[V]	Y336	8	0.19	1E-01
MAPK14	[R].HTDDEMTGYVATR.[W]	Y223	88	0.65	2E-02
PTK2	[R].YMEDSTYYK.[A]	Y662	92	0.22	2E-04
MAPK13	[R].HADAEMTGYVVTR.[W]	Y182	22	1.61	3E-01
SNRNP70	[R].EFEVYGPIKR.[I]	Y126	74	1.54	2E-02
NUCB1	[R].YEMLKEHER.[R]	Y179	6	0.05	3E-03
PTK2B	[R].YIEDEDYYKASVTRLPIK.[W]	Y579	16	4.61	4E-02
CTNNB1	[R].LHYGLPVVVK.[L]	Y489	8	1.82	1E-01
CALM2	[R].VFDKDGNGYISAAELR.[H]	Y148	43	2.56	9E-03
DOCK1	[K].GSVADYGNLMENQDLLGSPTPPPPPPHQR.[H]	Y1854	10	0.0001	1E-01
BCAR1	[R].VLPPEVADGGVVDSGVYAVPPPAER.[E]	Y456	7	0.43	1E-01

a PSM = Peptide-Spectrum Match

### Inhibition of CAMKK2 Leads to a Decline in Cell Proliferation, Invasion, Migration, and Colony Formation Activity of Gastric Adenocarcinoma Cells

**FIGURE 5:**
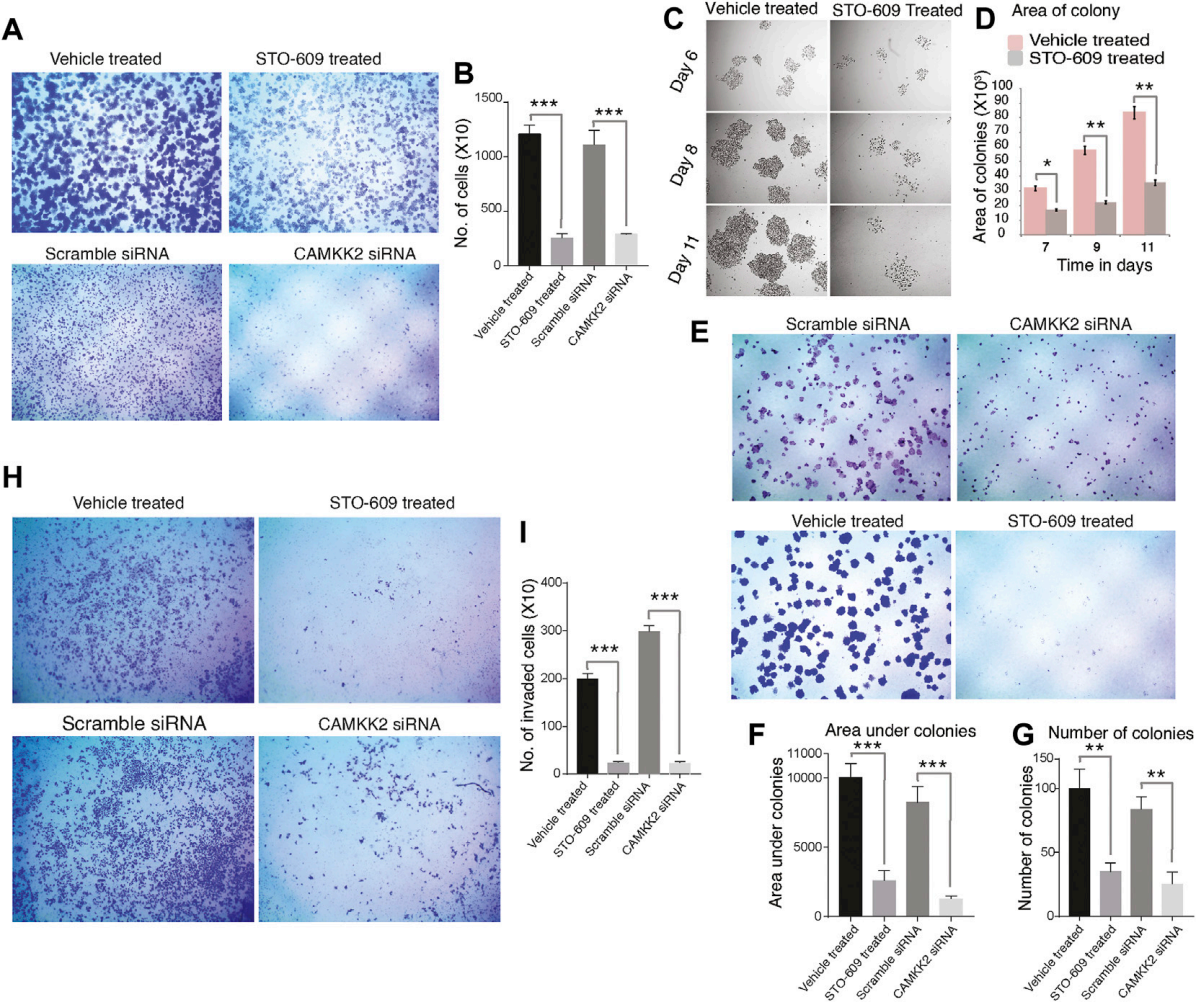
Inhibition or silencing of CAMKK2 leads to reduced cellular proliferation, invasion, migration, and colony formation in AGS cells. **(A)** Cellular proliferation of AGS cells upon CAMKK2 inhibition with STO-609 or silencing by CAMKK2 siRNA. Proliferated cells were stained with crystal violet and visualized under a microscope at 1× magnification. **(B)** Bar diagram depicting cellular proliferation activity of AGS cells upon CAMKK2 inhibition/silencing (***p < 0.001). **(C)** Representative images of colonies obtained from AGS cells upon STO-609 treatment; DMSO used as vehicle control. Photographs were taken at indicated time points at 10× magnification. **(D)** A bar diagram depicts colony size in AGS cells upon CAMKK2 inhibition (*p < 0.05, **p < 0.01). (e) Colony formation assay of AGS cells upon CAMKK2 inhibition by STO-609 or silencing with CAMKK2 siRNA. Colonies formed were stained with crystal violet to visualize under a microscope (1× magnification). **(F,G)** Results obtained from colony formation assay are represented as a bar chart showing colony number and area under colonies (**p < 0.01, ***p < 0.001). **(H)** Invasion assay to see the cellular invasion of AGS cells following treatment with STO-609, with DMSO used as vehicle control, and CAMKK2 siRNA or Scramble siRNA. Invasion assays were carried out in a transwell system using Matrigel-coated filters, and migrated cells to the lower chamber were counted. Cells were stained with crystal violet and visualized in a microscope (10× magnification). **(I)** Graphical representation of invasive ability of AGS cells upon CAMKK2 inhibition or silencing (***p < 0.001).

AGS cells were treated with STO-609 to inhibit CAMKK2, and DMSO was used as the control. We also performed siRNA-mediated silencing of CAMKK2 in AGS cells. Our results revealed a significant decrease in cellular proliferation upon inhibiting or silencing CAMKK2 in gastric cancer cells (Figures 5A,B). Next, we studied the colony-forming ability of AGS cells on CAMKK2 inhibition and silencing. We observed a significant reduction in the colony-forming ability of AGS cells in the presence of STO-609 when compared to the control cells (Figures 5C–G). We also found a reduction in colony-forming ability upon CAMKK2 silencing using siRNA. Our cell invasion assay result showed a significant decrease in the invasive ability of AGS cells upon inhibition or silencing of CAMKK2 (Figures 5H,I). Next, we have studied the effect of CAMKK2 inhibition on the cellular migration of AGS cells. We observed a significant reduction in the migratory property of AGS cells upon CAMKK2 inhibition using the scratch assay (Supplementary Figure S4A,B). These results collectively suggest that CAMKK2 inhibition or silencing has an anti-oncogenic effect on gastric cancer cells by regulating tyrosine phosphorylation. These results are in agreement with our previous studies, where we showed that inhibition of CAMKK2 leads to decreased cellular proliferation, invasion, migration, colony-formation ability, and cell cycle arrest in gastric cancer cells (Najar et al., 2021a; Najar et al., 2021b).

### Inhibition/Silencing of CAMKK2 Decreased Phosphorylation of PTK2, c-JUN, STAT3 Signaling in Gastric Cancer Cells

**FIGURE 6:**
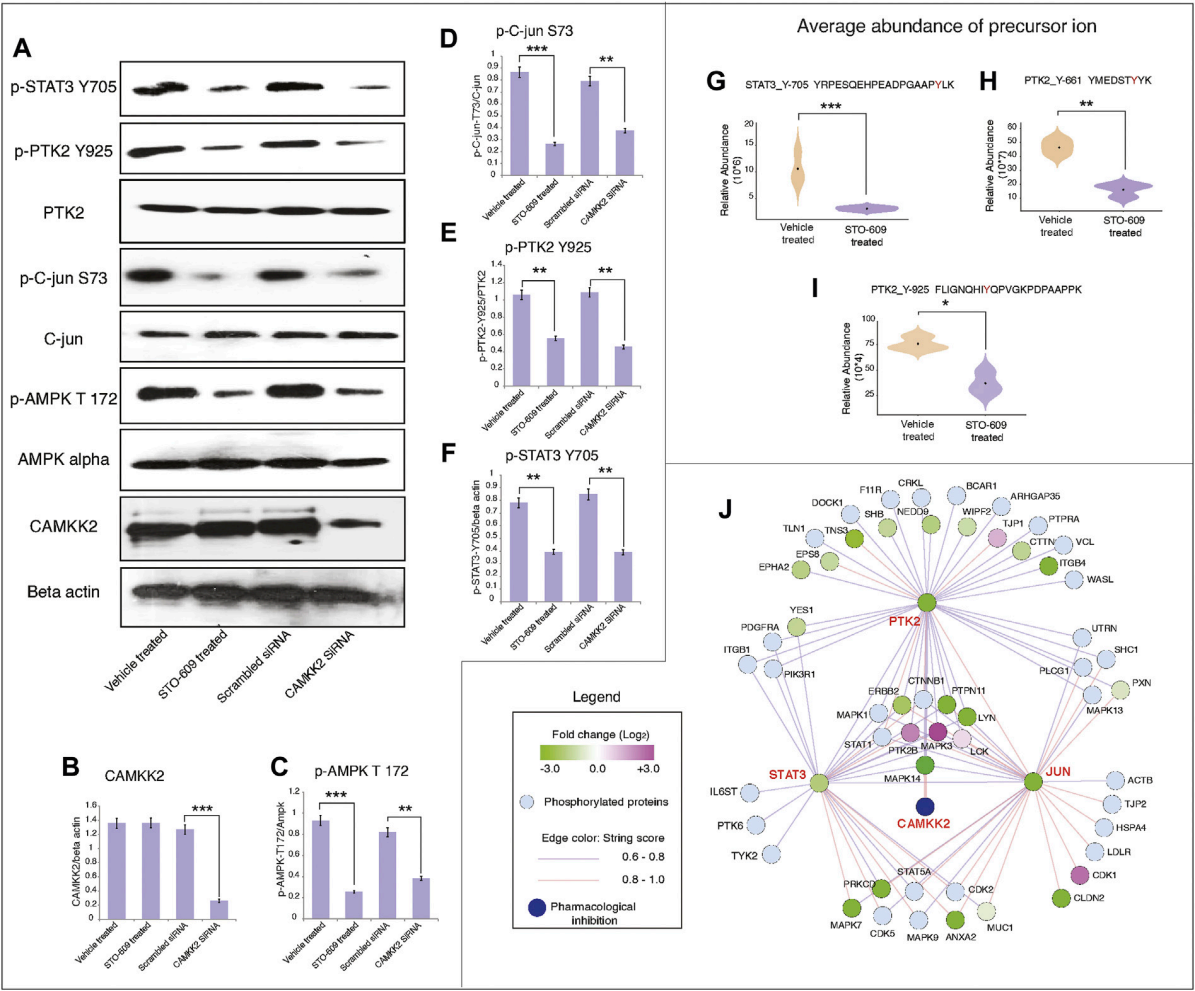
Inhibition of CAMKK2 decreased phosphorylation of PTK2/c-JUN and STAT3 signaling in gastric cancer cells. **(A)** Expression of indicated proteins in gastric cancer cells (AGS) on inhibition or silencing of CAMKK2 as indicated; β-Actin was used as a loading control. **(B)** Densitometry graph representing the expression of CAMKK2 for conditions depicted in **(A)** (***p < 0.001). **(C)** Densitometry graph representing the expression p-AMPK depicted in **(A)** (**p < 0.01, ***p < 0.001). **(D)** Densitometry graph representing the expression p-c-JUN depicted in **(A)** (**p < 0.01, ***p < 0.001). **(E)** Densitometry graph representing the expression p-PTK2 depicted in **(A)** (**p < 0.01). **(F)** Densitometry graph representing the expression p-STAT3 depicted in **(A)** (**p < 0.01). **(G–I)** Violin plot showing the expression of p-STAT3 and p-PTK2 from mass spectrometry data. **(J)** Network analysis of hypophosphoproteins in AGS cells upon CAMKK2 inhibition using the STRING (Version 11:0) database.

So far, our data suggest that inhibition of CAMKK2 decreases tyrosine phosphorylation of proteins involved in various oncogenic activities. We performed kinases and transcription factors enrichment of hypophosphorylated proteins using the eXpression2Kinases enrichment tool (X2K Web’s tool) (Clarke et al., 2018) to predict the kinases and transcription factors regulated by CAMKK2. We observed enrichment of different kinases like ERK1, ERK2, MAPK14, and GSK3B; transcription factors like RUNX1, ZBTB7A, and STAT3; and intermediate proteins like SMAD1, SMAD2, HDAC3, CDK1, and JUN (Figure 4D). We also observed decreased phosphorylation of PTK2 upon inhibition of CAMKK2, which is consistent with earlier reports that showed PTK2 regulates phosphorylation of STAT3 (Thakur et al., 2015). Our mass spectrometry data revealed hypophosphorylation of PTK2 at Y925, Y661, and STAT3 at Y705 (Figures 6G–I and Supplementary Figure S5A1–C3); this was further confirmed by the western blot analysis. Our western blot data revealed decreased phosphorylation of PTK2 at Y925, p-c-JUN at S73, STAT3 at Y705, and AMPK at T172 upon both inhibition and silencing of CAMKK2 in gastric cancer (Figures 6A–F). These results are in concordance with the mass spectrometry results (Supplementary Figure S5A1–C3). The interactome analysis for hypophosphorylated proteins on CAMKK2 inhibition showed a strong interaction with each other with strong clusters with PTK2, JUN, and STAT3 (Figure 6J). The Ingenuity Pathway analysis also revealed different networks such as JUN, STAT3, and VEGF which was in concordance with the interactome analysis (Supplementary Figure S2B–D). These results suggest that inhibition/silencing of CAMKK2 decreases gastric cancer transformation by regulating the activity of PTK2/JUN/STAT3 signaling.

## Discussion

Gastric cancer is the fifth most common cancer and the third leading cause of cancer-related deaths worldwide (Parkin, 2004; Bray et al., 2018). Pathogenesis of GC converges on dysregulation of the common signaling pathways as a direct or secondary consequence in activation of proto-oncogenes and inactivation of tumor-suppressor genes (Wu et al., 2010). Deregulation of tyrosine phosphorylation has been reported in different cancers, such as GC (Hitosugi et al., 2009; Hitosugi et al., 2011; Da Costa et al., 2018; Liu et al., 2019). Tyrosine kinases phosphorylate their substrates at tyrosine residue. Multiple reports show tyrosine kinases are involved in S/T phosphorylation through intermediate kinases (Bhat et al., 2018; Pinto et al., 2018; Sahu et al., 2018). CAMKK2 belongs to the STK family and is reported to be overexpressed in different types of cancers, such as gastric cancer. However, its role in regulating tyrosine phosphorylation is not prominent. Studying tyrosine phosphoproteome using mass spectrometry has gained importance in cancer research, drug discovery, and molecular signaling. In this study, we evaluated the regulation of tyrosine phosphorylation by CAMKK2 in gastric cancer by mass spectrometry–based phosphotyrosine proteomic analysis. To study the role of CAMKK2 in phosphotyrosine signaling, we used STO-609–mediated inhibition of CAMKK2 in gastric cancer cells. It is the first high-throughput study to profile CAMKK2-regulated phosphotyrosine proteome in gastric cancer.

Ephrin type-A receptor 2 (EPHA2) belongs to the ephrin receptor subfamily of the protein tyrosine kinase family. In normal conditions, EPHA2 regulates migration, integrin-mediated adhesion, proliferation, and differentiation of cells. EPHA2 is reported to be overexpressed in gastric cancer (Hong et al., 2018) and colorectal cancer (Hong et al., 2018) and is involved in the increased invasion, migration, and prognosis of colorectal cancer cells (Hong et al., 2018). Targeting EPHA2 has decreased cell cycle progression and growth of basal-like/triple-negative breast cancers (Song et al., 2017). In our data, we have observed a decrease in phosphorylation of EPHA2 at Y588, Y575, Y594, and Y772 upon inhibition of CAMKK2 in GC cells. Phosphorylation at Y588, Y575, and Y594 of EPHA2 are required for EPHA2-dependent RAC1 activation and cell migration (Fang et al., 2008). Ephrin-2 (EFNB2) belongs to the ephrin receptor subfamily of the protein tyrosine kinase family. Studies have shown that ephrins regulate tumor cells’ invasion, migration, and angiogenesis (Castellvi et al., 2006). EFNB2 is expressed abnormally high in multiple cancers, such as gastric cancer (Kataoka et al., 2002; Tachibana et al., 2007; Yavrouian et al., 2008; Li et al., 2015; Li et al., 2017). Our data indicated decreased phosphorylation of EFNB2 at Y304 upon inhibition of CAMKK2. Hyperphosphorylation of EFNB2 at Y304 is one of the most common incidences of multiple cancer (Nakada et al., 2010; Zhu et al., 2020). Its increased phosphorylation is a driver in EFNB2-dependent cancer development (Bai et al., 2012). Apart from receptor tyrosine kinases, we have also identified hypophosphorylation of non-receptor tyrosine kinases such as PTK2, protein tyrosine kinase 2-beta (PTK2B), tyrosine protein kinase Lck (LCK), and tyrosine protein kinase Lyn (LYN) upon inhibition of CAMKK2. The PTK genes make up the largest family of oncogenes, and their dysregulation is involved in cancer development (Xi et al., 2012; Sugiyama et al., 2019). PTK2 is reported to take part in various singling pathways that promote cancer growth and metastasis through kinase-dependent control of cell motility (Mitra et al., 2005), invasion (Shibue et al., 2012), cell survival (Cance and Golubovskaya, 2008; Frisch et al., 2013), and transcriptional events promoting epithelial to mesenchymal transition (EMT) (Li et al., 2011). Overexpression of PTK2 has been reported in GC tissue when compared to adjacent normal tissue (Luo et al., 2020). Our data revealed a decrease in phosphorylation of PTK2 at Y661 and Y925 upon inhibition of CAMKK2. The tyrosine protein kinase Mer precursor (MERTK) is a tyrosine kinase that regulates many physiological processes, such as cell survival, migration, differentiation, and phagocytosis of apoptotic cells. MERTK has been reported to be overexpressed in multiple cancers along with gastric cancer (Yi et al., 2017). MERTK activation is reported to phosphorylate p38, ERK1/2, GSK3a/b, AKT, AMPK, STAT5, CHK2, PTK2, and STAT6 (Schlegel et al., 2013; Tworkoski et al., 2013). We have observed a 2-fold decrease in phosphorylation of MERTK at Y749 and its downstream substrate PTK2 at Y662, and STAT3 at Y737 and Y705. Inhibition of STAT3 at its N-terminal domain has shown promising anticancer activity in breast cancer cells (Timofeeva et al., 2007). Another interesting protein non-receptor tyrosine protein kinase TYK2 (TYK2) has been reported as overexpressed in prostate (Ide et al., 2008; Santos et al., 2015), ovarian (Silver et al., 2004), cervical (Zhu et al., 2009), and breast (Song et al., 2008) cancers. Studies have shown hyperactivation of TYK2 phosphorylates STAT1/3/5 (Sanda et al., 2013). Another study shows TYK2 phosphorylates STAT3 at Y705 and Y640 (Mori et al., 2017). Our data show a decrease in the phosphorylation of TYK2 at Y292 and STAT3 at Y705 and Y737 on inhibition of CAMKK2 in gastric cancer.

In addition to kinases, we also identified decreased phosphorylation of non-kinase proteins upon inhibition of CAMKK2 in gastric cancer. These proteins include plakophilin-4 (PKP4), annexin A2 (ANXA2), nucleobindin-1 (NUCB1), cofilin-1 (CFL1), enhancer of filamentation 1 (NEDD9), Erbin (ERBIN), protein arginine N-methyltransferase 1 (PRMT1), and ATP-dependent RNA helicase DDX3X (DDX3X). ANXA2 is reported to be overexpressed in esophageal cancer and is involved in cancer progression by activating the MYC-HIF1A-VEGF axis (Droz et al., 1985). It is reported that ANXA2 enhances STAT3 activation and promotes proliferation and invasion of breast cancer cells (Yuan et al., 2017). Another report showed ANXA2 binds to STAT3 and promotes epithelial to mesenchymal transition in breast cancer cells (Wang et al., 2015). Increased tyrosine phosphorylation of annexin A2 promotes proliferation, invasion, and STAT3 phosphorylation in breast cancer cells (Wang et al., 2012). Interestingly, we have found decreased phosphorylation of ANXA2 at Y715 and a decrease in phosphorylation of STAT3 upon inhibition of CAMKK2 in gastric cancer cells. The eXpression2Kinases analysis revealed enrichment of c-JUN, an upstream activator of STAT3 from hypophosphorylated proteins upon inhibition of CAMKK2. Our data revealed decreased phosphorylation of PTK2, c-JUN, and STAT3 upon inhibition and siRNA-mediated silencing of CAMKK2 in gastric cancer cells. This indicates that CAMKK2-mediated signaling may orchestrate through PTK2/JUN/STAT3 signaling in gastric cancer. Our work provides a scaffold for future studies to investigate the role of CAMKK2 in regulating tyrosine signaling in gastric cancer.

## Conclusion

Our data provide a glimpse of CAMKK2-regulated tyrosine signaling in gastric cancer cells, which involves the PTK2/JUN/STAT3 axis. This study will serve as a road map for CAMKK2 and its downstream signaling in gastric cancer and offer a potential starting point for further mechanistic studies. Our phosphoproteomic data and bioinformatics analyses lead to the identification of both kinases and non-kinases regulated by CAMKK2, which can serve as potential therapeutic targets for gastric cancer. A more detailed understanding about the role of CAMKK2 in activation of the tyrosine phosphoproteome and, in particular, PTK2/JUN/STAT3 signaling in gastric cancer is needed, in addition to functional studies in preclinical models, which is beyond the scope of this article.

## Data Availability

The datasets presented in this study can be found in online repositories. The names of the repository/repositories and accession number(s) can be found below: https://www.ebi.ac.uk/pride/, PXD032001.

## Supplementary Material

Supplementary Figure 1 CAMKK2 inhibition regulates the phosphorylation of proteins involved in different pathologies. **(A)** Interactome analysis of proteins identified and expression of their p-Y upon inhibition of CAMKK2 in gastric cancer. **(B)** Bar graph showing pathological conditions of hypophosphorylated non-kinase proteins are involved in, Metascape was used for data analysis. **(C)** Bar graph showing pathological conditions of hypophosphorylated kinase are involved in, Metascape was used for data analysis.

Supplementary Figure 2 CAMKK2 inhibition decreases the phosphorylation of proteins involved in cancer development. **(A)** Heat maps highlight significant downstream biological processes enriched for altered phospho-Y-proteome upon inhibition of CAMKK2 using Ingenuity Pathway analysis. **(B**–**D)** The top-scoring IPA protein networks indicated upon CAMKK2 inhibition in gastric cancers.

Supplementary Figure 3 Identification of novel kinases regulated by CAMKK2 in gastric cancer cells. **(A)** Interactome and GO analysis of novel kinases regulated by CAMKK2 in gastric cancer cells. Metascape tool with STRING, BioGrid, OmniPath databases were used for data analysis. **(B)** Bar graph showing pathological conditions in which novel kinases are involved, Metascape was used for data analysis.

Supplementary Figure 4 CAMKK2 inhibition decreases cell migration of gastric cancer cells. **(A)** Wound migration assay was carried out using gastric cancer cell line AGS upon treatment with STO-609 and vehicle control. Cells were imaged at 4× magnification. **(B)** Graphical representation of the migratory ability of AGS cells treated with STO-609 compared to vehicle control (*p<0.05, **p<0.01).

Supplementary Figure 5 Representative MS/MS spectra of peptides of up and downregulated proteins in gastric cancer cells (AGS) upon CAMKK2 inhibition. **(A1, B1 and C1)** Represents base peak of the precursor ions between STO-609 treated and vehicle-treated of represented peptides in gastric cancer cells. **(A2, B2 and C2)** Represents the MS/MS spectra of the represented phosphopeptides. **(A3, B3 and C3)** Violin plot showing the expression of represented peptides from mass spectrometry data.

Supplementary Figure 6 Venn diagram of proteins identified between the control and STO-609 treated in AGS cells. **(A)** Venn diagram showing the protein identifications between triplicates in control sample. **(B)** Venn diagram showing the protein identifications between triplicates in STO-609 treated sample. **(C)** Venn diagram shows the protein identifications between control and STO-609 treated samples across the triplicates.

Supplementary Table S1 List of phosphopeptides identified and quantified in LFQ-based quantitative phosphoproteomic approach in gastric cancer cells (AGS) upon CAMKK2 inhibition.

Supplementary Table S2 List of differentially phosphorylated peptides upon inhibition of CAMKK2 in gastric cancer cells (AGS).

## Abbreviations

CAMKK2: calcium/calmodulin‐dependent protein kinase kinase 2; CDK1: cyclin‐dependent kinase 1; DMSO: dimethyl sulfoxide; ERK1: extracellular signal–regulated protein kinase 1; FBS: fetal bovine serum; PTK2: focal adhesion kinase 1; STAT3: signal transducer and activator of transcription 3; c-JUN: transcription factor AP-1; TEABC: triethylammonium bicarbonate buffer; LFQ: label-free quantification.
