# Supplementary figures and images for: Tyrosine Phosphorylation Profiling Revealed the Signaling Network Characteristics of CAMKK2 in Gastric Adenocarcinoma
Source: Front Genet. 2022 May 13;13:854764. doi: 10.3389/fgene.2022.854764 (PMC9136244; doi:10.3389/fgene.2022.854764)

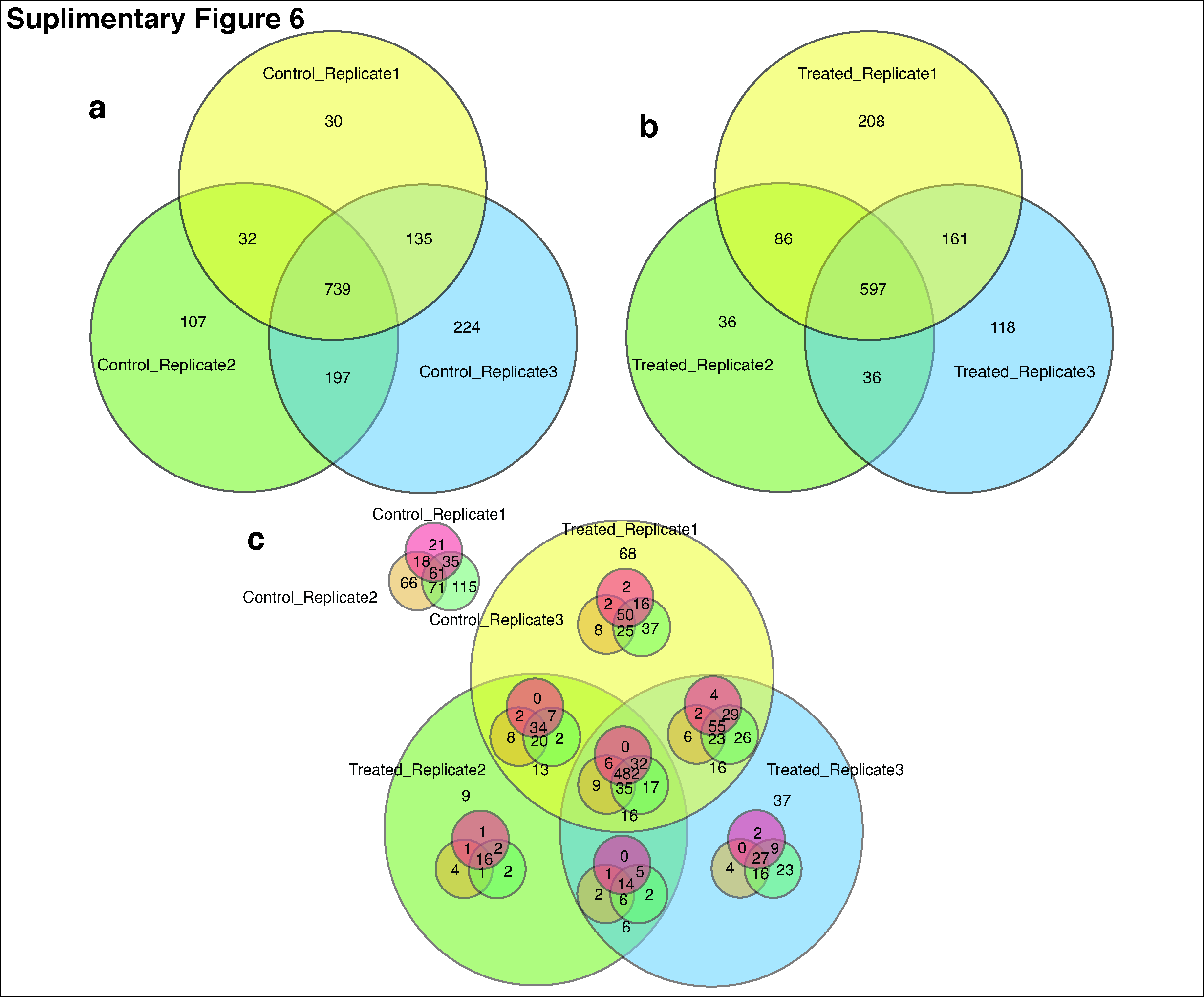

Supplement: Supplementary file 1 [file Image6.TIF]

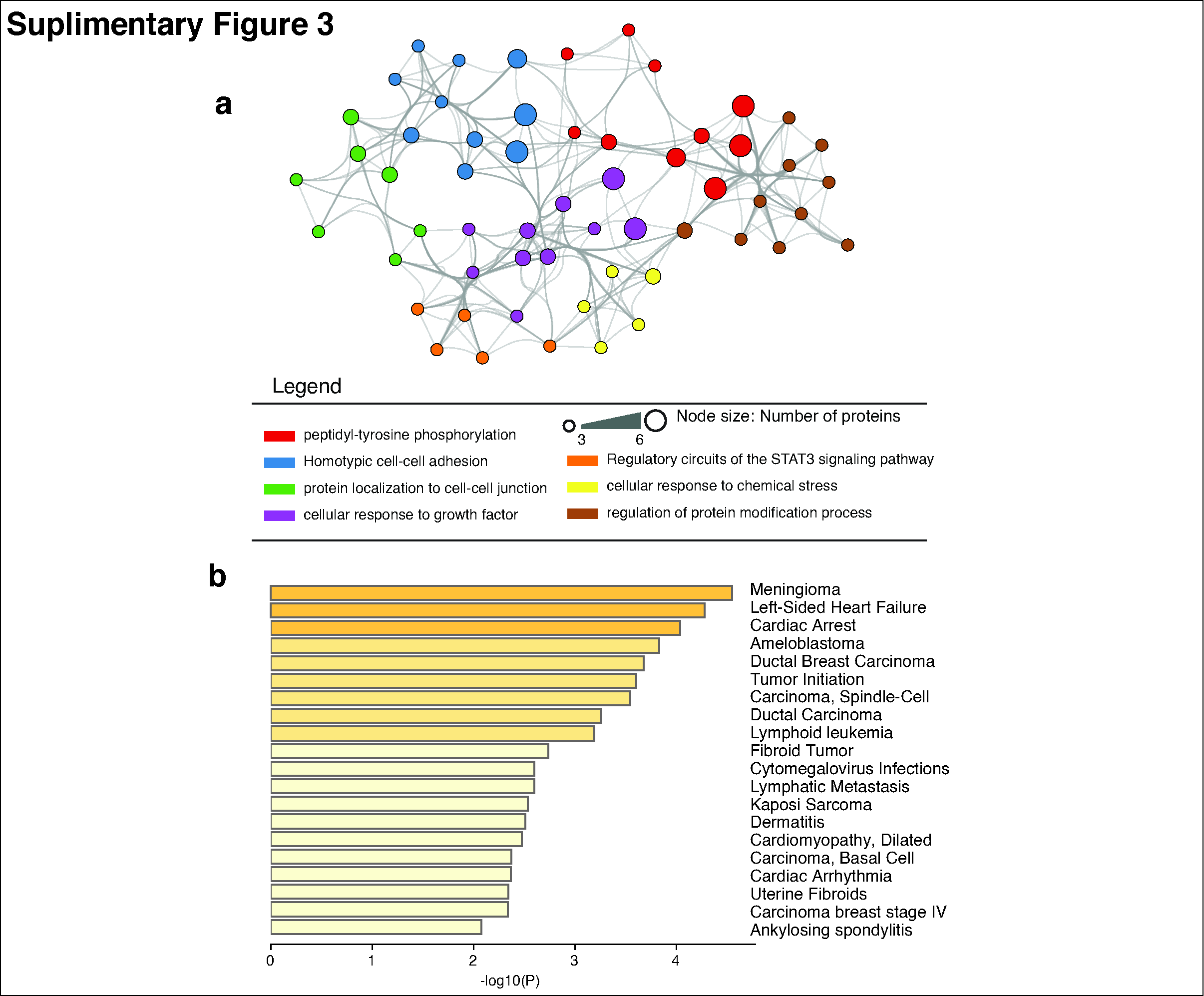

Supplement: Supplementary file 3 [file Image3.TIF]

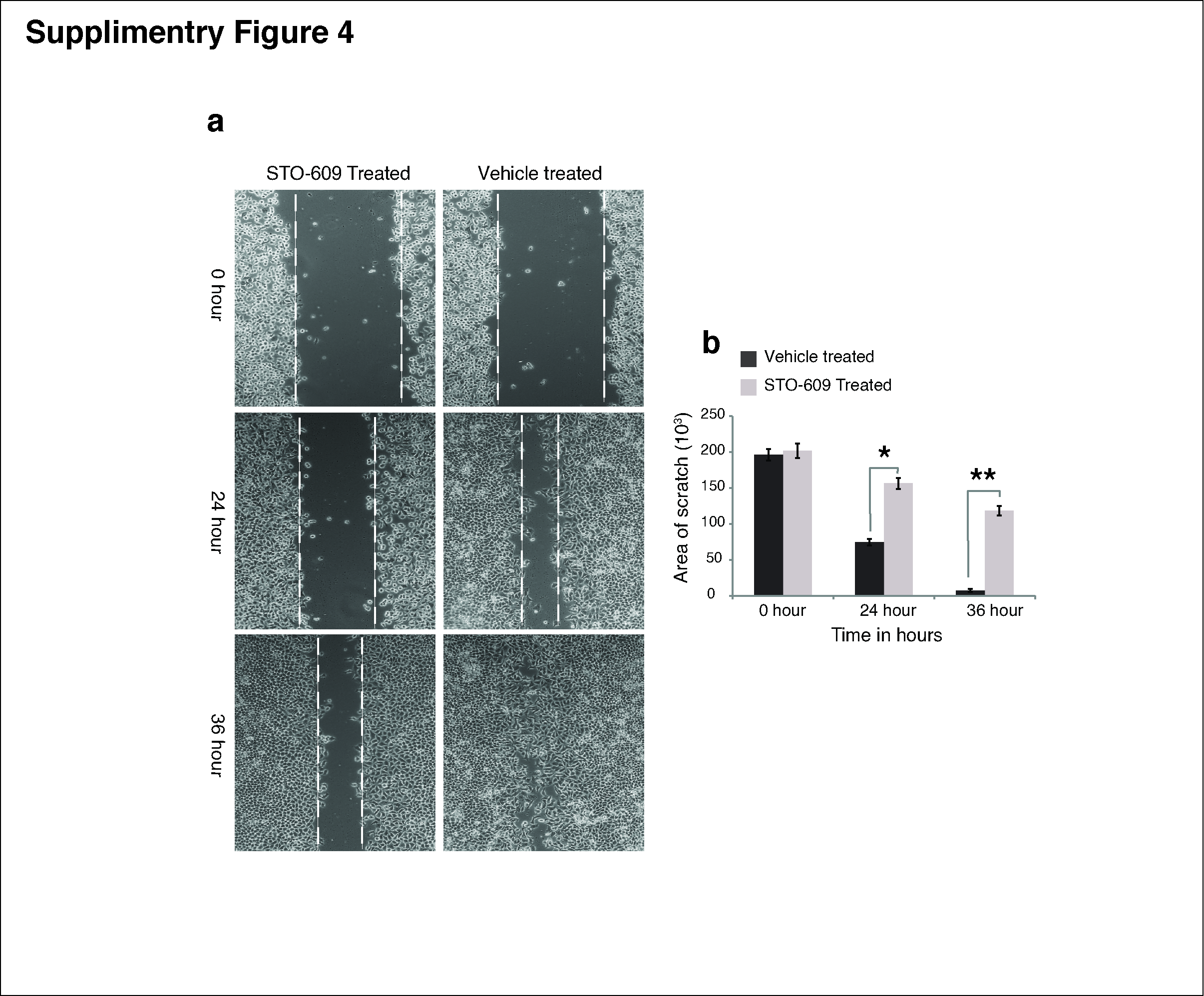

Supplement: Supplementary file 4 [file Image4.TIF]

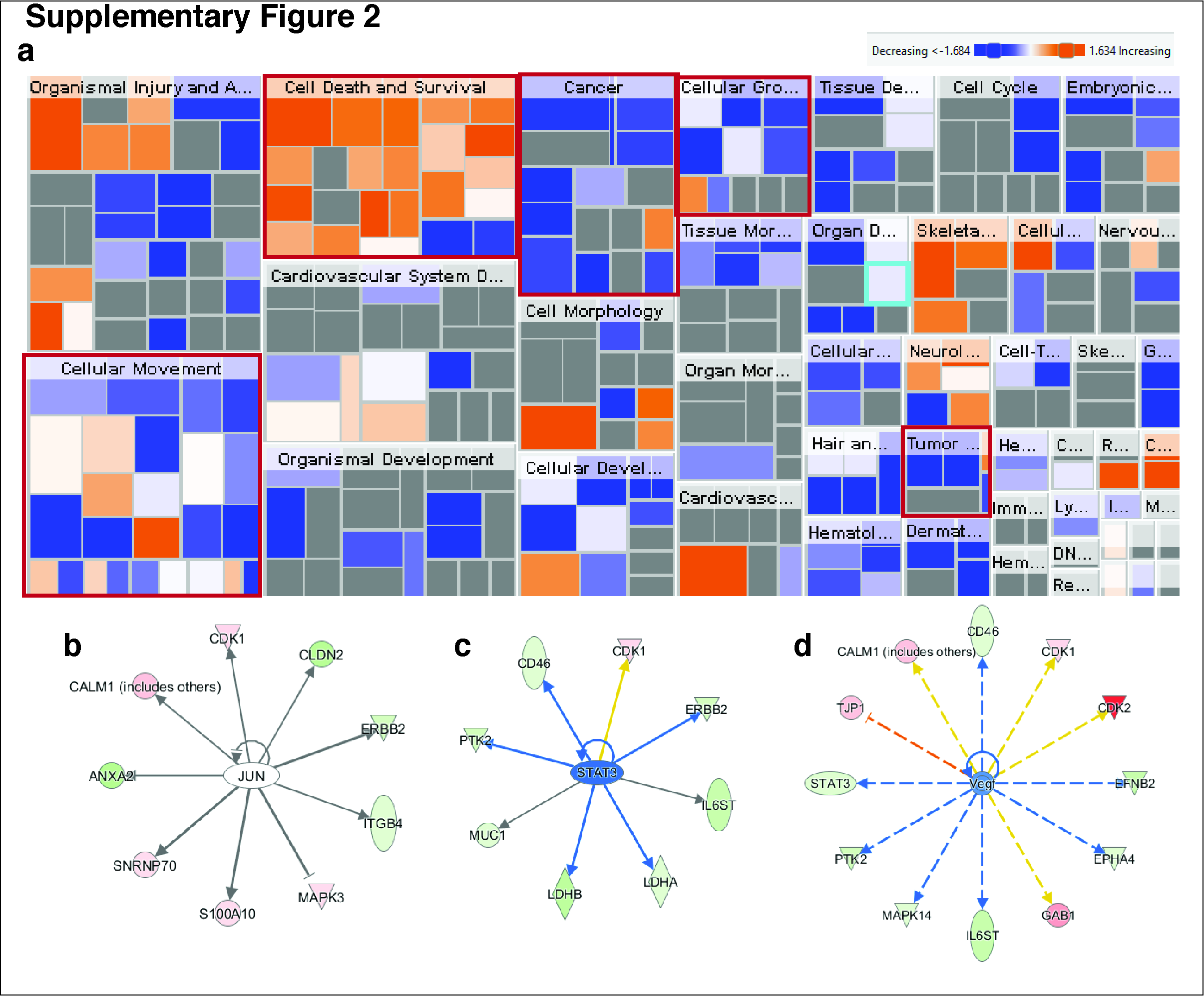

Supplement: Supplementary file 6 [file Image2.TIF]

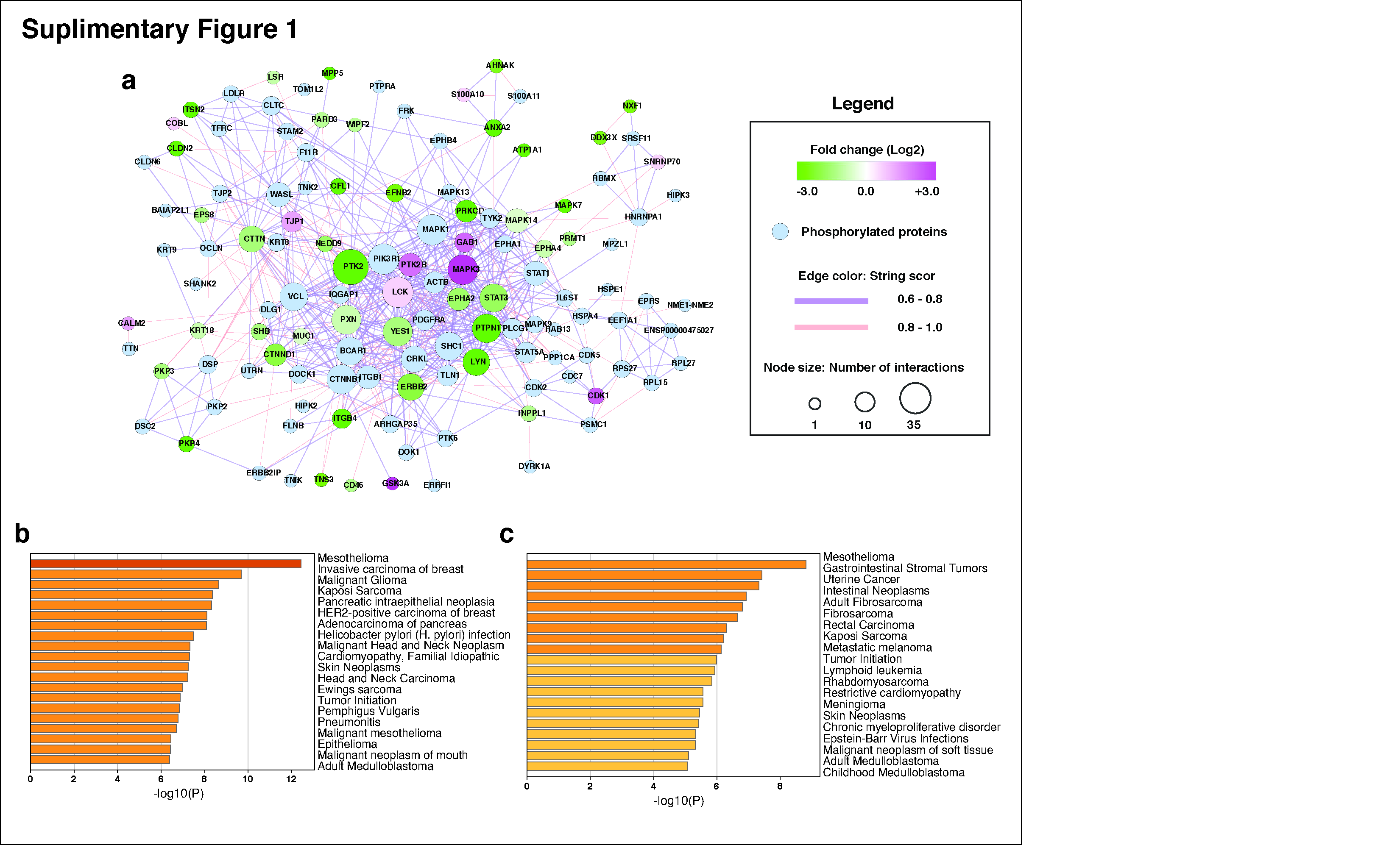

Supplement: Supplementary file 7 [file Image1.TIF]

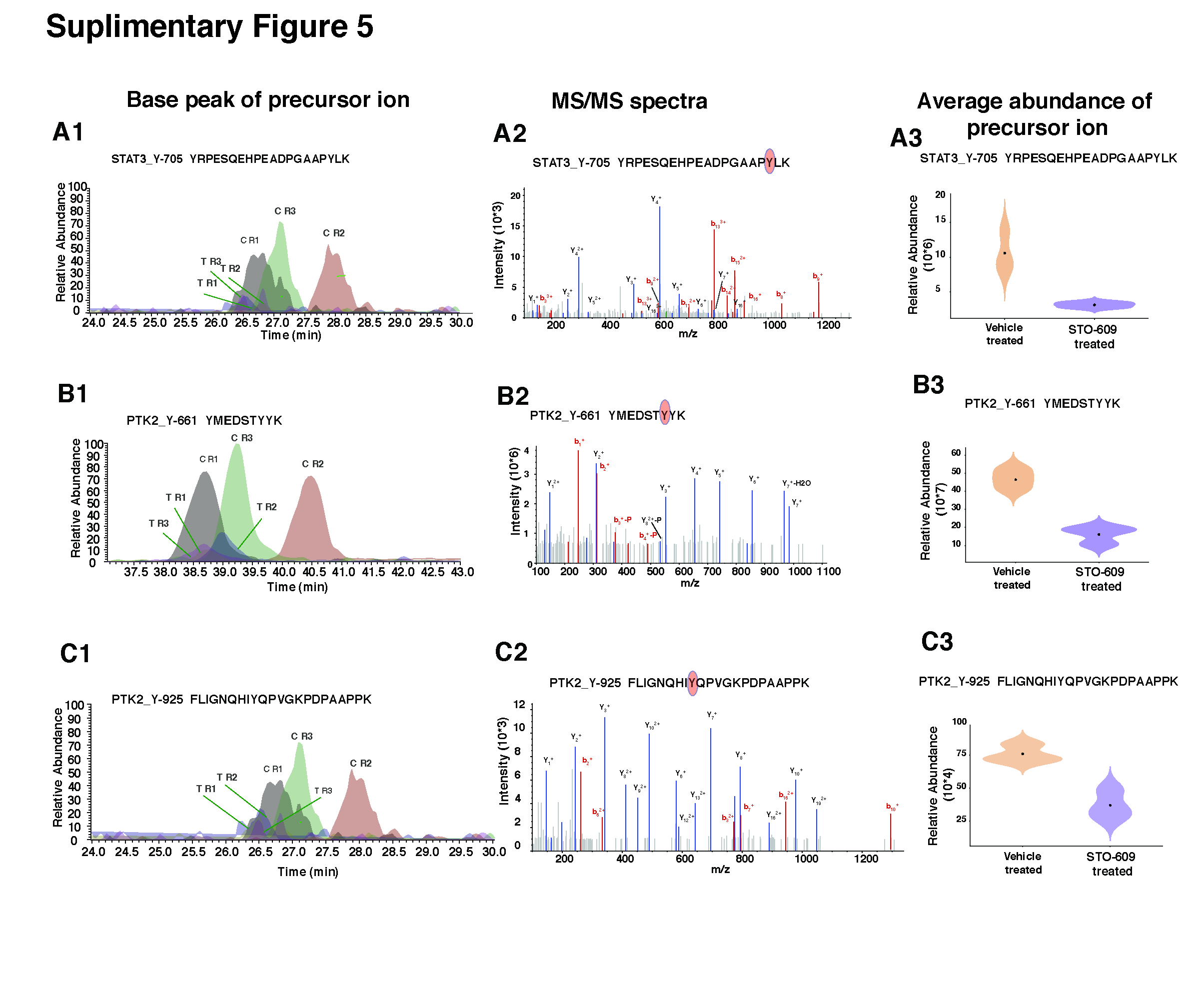

Supplement: Supplementary file 8 [file Image5.TIF]
