# Supplementary material for: Tyrosine Phosphorylation Profiling Revealed the Signaling Network Characteristics of CAMKK2 in Gastric Adenocarcinoma
Source: Front Genet. 2022 May 13;13:854764. doi: 10.3389/fgene.2022.854764 (PMC9136244; doi:10.3389/fgene.2022.854764)
Supplement: Supplementary file 2 [file Table1.PDF]

Najar et al; Supplementary Table S1: List of phosphopeptides identified and quantified in LFQ based quantitative phosphoproteomic approach in gastric cancer cells (AGS) upon CAMKK2 inhibition.

Accession	Gene	Gene ID	Protein	Annotated Sequence	Modifications site	# PSMs	FC(T/C)	p-Value
NP_001164185.1	BCAR1	9564	breast cancer anti-estrogen resistance protein 1 isoform 1	[R].RPGPGTLYDVPR.[E]	Y433	17	2.228450082	0.068326087
NP_001341605.1	PRKCD	5580	protein kinase C delta type isoform a	[R].RSDSASSEPVGIYQGFEK.[K]	Y332	2	0	8.71579E-06
NP_001341605.1	PRKCD	5580	protein kinase C delta type isoform a	[R].RSDSASSEPVGIYQGFEKK.[T]	Y332	23	2.21003762	0.27040664
XP_011510318.1	PKP4	8502	plakophilin-4 isoform X1	[R].SAVSPDLHITPIYEGR.[T]	Y415	7	0.576200417	0.067661416
XP_011540550.2	PLEKHN1	84069	pleckstrin homology domain-containing family N member 1 isoform X1	[R].SCSSGPAGPYLLSK.[K]	Y678	30	1.815206609	0.145923263
NP_004422.2	EPHA2	1969	ephrin type-A receptor 2 isoform 1 precursor	[K].SEQLKPLKTYVDPHTYEDPNQAVLK.[F]	Y588	56	0.469038402	0.018861003
NP_004422.2	EPHA2	1969	ephrin type-A receptor 2 isoform 1 precursor	[K].SEQLKPLKTYVDPHTYEDPNQAVLK.[F]		274	0.638813048	0.050277053
NP_003944.1	MPZL1	9019	myelin protein zero-like protein 1 isoform a precursor	[K].SESVVYADIR.[K]	Y263	2	0.817540342	0.390037269
NP_004422.2	EPHA2	1969	ephrin type-A receptor 2 isoform 1 precursor	[R].QSPEDVYFSK.[S]	Y575	18	0.617530982	0.055220871
NP_004422.2	EPHA2	1969	ephrin type-A receptor 2 isoform 1 precursor	[R].QSPEDVYFSKSEQLKPLK.[T]		78	1.391029007	0.040720135
NP_001158886.1	LDHA	3939	L-lactate dehydrogenase A chain isoform 3	[K].QVVESAYEVIK.[L]	Y268	2	0.362504455	0.047912142
NP_063937.2	GSK3A	2931	glycogen synthase kinase-3 alpha	[K].QLVRGEPNVSYICSR.[Y]	S278	198	15.99737533	0.011440071
NP_001347.3	DDX3X	1654	ATP-dependent RNA helicase DDX3X isoform 1	[R].RKQYPISLVLAPTR.[E]	Y266	12	0.917320956	0.554987796
NP_001135865.1	NEDD9	4739	enhancer of filamentation 1 isoform 3	[R].RHQSLSPNHPPPQLGQSVGSQNDAYDVPR.[G]	Y317	24	0.898602421	0.690167735
NP_112420.1	HNRNPA1	3178	heterogeneous nuclear ribonucleoprotein A1 isoform b	[R].SSGPYGGGGQYFAKPR.[N]		132	1.988246577	0.056176542
XP_016863455.1	GAB1	2549	GRB2-associated-binding protein 1 isoform X1	[K].SSGSGSSVADERVDYVVVDQQK.[T]	Y703	18	4.940580648	0.016563757
NP_991403.1	LSR	51599	lipolysis-stimulated lipoprotein receptor isoform 2	[R].SSSAGGQGSYVPLLR.[D]		41	1.215176979	0.242266914
NP_000215.1	KRT18	3875	keratin, type I cytoskeletal 18	[R].STFSTNYR.[S]	Y13	26	0.529435908	0.034734437
NP_991403.1	LSR	51599	lipolysis-stimulated lipoprotein receptor isoform 2	[R].SRDPHYDDFR.[S]	Y551	48	0.8302318	0.493555082
XP_016865074.1	PIK3R1	5295	phosphatidylinositol 3-kinase regulatory subunit alpha isoform X1	[K].SREYDRLYEEYTR.[T]	Y467	21	1.110733778	0.661083759
XP_024305828.1	PEAK1	79834	pseudopodium-enriched atypical kinase 1 isoform X1	[K].SSAIRYQEVWTSSTSPR.[Q]	Y531	23	4.641459761	0.005205982
XP_011510318.1	PKP4	8502	plakophilin-4 isoform X1	[K].STTNYVDFYSTK.[R]	Y1168	40	0.644910948	0.047285937
NP_001002857.1	ANXA2	302	annexin A2 isoform 2	[K].SYSPYDMLESIR.[K]	Y238	11	0.092471918	0.000890654
NP_001002857.1	ANXA2	302	annexin A2 isoform 2	[K].SYSPYDMLESIRK.[E]	Y238	34	0.925755452	0.72332633
NP_001243211.1	KRT8	3856	keratin, type II cytoskeletal 8 isoform 1	[K].SYKVSTSGPR.[A]	Y38	48	0.881818236	0.74974335
NP_001393.1	EEF1A1	1915	elongation factor 1-alpha 1	[K].STTTGHLIYK.[C]	Y29	62	1.162119695	0.380377708
XP_016865974.1	ICK	22858	serine/threonine-protein kinase ICK isoform X1	[R].SKPPYTDYVSTR.[W]		31	0.296435612	0.02176451
XP_011512144.1	PCDH7	5099	protocadherin-7 isoform X1	[K].SKQPLYSSIVTVEASKPNGQR.[Y]		43	3.378651972	0.093015226
XP_024306673.1	SRSF11	9295	serine/arginine-rich splicing factor 11 isoform X1	[R].SKTPPKSYSTAR.[R]	T325	38	1.723095406	0.151802362
NP_002341.1	LYN	4067	tyrosine-protein kinase Lyn isoform A	[R].SLDNGGYYISPR.[I]	Y194	8	0.799257845	0.485988254
NP_001078927.1	CTNND1	1500	catenin delta-1 isoform 1ABC	[K].SLDNNYSTPNER.[G]	Y904	20	0.168746694	0.000906702
NP_001372.1	DOK1	1796	docking protein 1 isoform a	[K].SHNSALYSQVQK.[S]		45	0.564330692	0.080746134
NP_061330.2	BAIAP2L1	55971	brain-specific angiogenesis inhibitor 1-associated protein 2-like protein 1	[R].SNVVRKDYDTLSK.[C]	Y274	17	1.698848281	0.106129743
NP_001164563.1	CLDN2	9075	claudin-2	[R].SNYYDAYQAQPLATR.[S]	Y198	80	0.103151984	0.001263014
NP_001037.1	SLC12A2	6558	solute carrier family 12 member 2 isoform 1	[K].SPGTKDVVVSVEYSK.[K]	Y/S	3	#DIV/0!	#DIV/0!
NP_003944.1	MPZL1	9019	myelin protein zero-like protein 1 isoform a precursor	[K].SLPSGSHQGPVIYAQLDHSGGHHSDK.[I]		50	0.510657218	0.025292322
NP_003944.1	MPZL1	9019	myelin protein zero-like protein 1 isoform a precursor	[K].SLPSGSHQGPVIYAQLDHSGGHHSDKINK.[S]	Y241	53	1.340994555	0.347700985
XP_024308698.1	ITSN2	50618	intersectin-2 isoform X1	[K].LIYLVPEK.[Q]	Y553	10	0.957333099	0.903977156
XP_005262892.1	FAT1	2195	protocadherin Fat 1 isoform X1	[K].LNKNIYSDIPPQVPVRPISYTPSIPSDSR.[N]	S/Y	7	0.883804026	0.902160142
NP_003944.1	MPZL1	9019	myelin protein zero-like protein 1 isoform a precursor	[K].INKSESVVYADIR.[K]	Y263	22	2.102767862	0.21271876

Najar et al; Supplementary Table S1: List of phosphopeptides identified and quantified in LFQ based quantitative phosphoproteomic approach in gastric cancer cells (AGS) upon CAMKK2 inhibition.

NP_001002857.1	ANXA2	302	annexin A2 isoform 2	[K].LSLEGDHSTPPSAYGSVK.[A]		407	2.096123191	0.012998678
NP_003019.2	SHB	6461	SH2 domain-containing adapter protein B	[K].LPQDDDRPADEYDQPWEWNR.[V]	Y336	8	0.192746293	0.103497464
NP_001317366.1	PTPN11	5781	tyrosine-protein phosphatase non-receptor type 11 isoform 3	[K].IQNTGDYYDLYGGEK.[F]		56	0.719070404	0.009220017
NP_003861.1	IQGAP1	8826	ras GTPase-activating-like protein IQGAP1	[K].LQQTYAALNSK.[A]	Y1510	4	5.442826704	0.078027593
NP_001895.1	CTNNB1	1499	catenin beta-1 isoform 1	[R].LHYGLPVVVK.[L]	Y489	8	1.820653428	0.143866796
NP_005223.4	EPHA1	2041	ephrin type-A receptor 1 precursor	[R].LLDDFDGTYETQGGKIPIR.[W]		131	1.098360045	0.861717626
XP_024306409.1	ERBB2	2064	receptor tyrosine-protein kinase erbB-2 isoform X1	[R].LLDIDETEYHADGGKVPIK.[W]	Y923	46	0.236155347	0.00937668
XP_016881449.1	YES1	7525	tyrosine-protein kinase Yes isoform X1;ref|XP_024302814.1	[R].LIEDNEYTAR.[EQ]	Y426	78	0.718889192	0.198951777
XP_011510318.1	PKP4	8502	plakophilin-4 isoform X1	[R].NNYALNTTATYAEPYRPIQYR.[V]		143	0.606121159	0.160040934
XP_011510318.1	PKP4	8502	plakophilin-4 isoform X1	[R].NNYALNTTATYAEPYRPIQYR.[V]	Y470; Y/T	9	0	1.71035E-05
XP_024302814.1	LCK	3932	tyrosine-protein kinase Lck isoform X1	[R].NLDNGGFYISPR.[I]	Y250	30	1.582490362	0.015231
XP_024305828.1	PEAK1	79834	pseudopodium-enriched atypical kinase 1 isoform X1	[K].NAIKVPIVINPNAYDNLAIYK.[S]	Y635	21	1.428567023	0.797322871
NP_004482.4	ARHGAP35	2909	rho GTPase-activating protein 35	[R].NEEENIYSVPHDSTQGK.[I]	Y1105	102	0.947247084	0.826374292
XP_011517854.2	KIAA1217	56243	sickle tail protein homolog isoform X1	[R].NEGFYADPYLYHEGR.[M]	Y467	16	0.430522693	0.0363745
NP_112420.1	HNRNPA1	3178	heterogeneous nuclear ribonucleoprotein A1 isoform b	[R].NQGGYGGSSSSSSYGSGR.[R]	Y357	26	0.432371831	0.149161846
NP_005198.1	CRKL	1399	crk-like protein	[R].NSNSYGIPEPAHAYAQPQTTTPLPAVSGSPGAAITPLPSTQNGPVFAK.[A]		145	1.41030236	0.776755876
NP_065804.2	CASKIN2	57513	caskin-2 isoform a	[R].NTYNQTALDIVNQFTTSQASR.[E]		20	0	0.353707828
NP_005834.4	STAM2	10254	signal transducing adapter molecule 2	[K].LVNEAPVYSVYSK.[L]		6	#DIV/0!	#DIV/0!
NP_001135865.1	NEDD9	4739	enhancer of filamentation 1 isoform 3	[K].LYQVPNPQAAPR.[D]	Y92	21	1.354762572	0.53074068
XP_006724039.1	DYRK1A	1859	dual specificity tyrosine-phosphorylation-regulated kinase 1A isoform X1	[R].IYQYIQSR.[F]	Y321	111	1.260344584	0.482420111
XP_011512733.1	ANKS1A	23294	ankyrin repeat and SAM domain-containing protein 1A isoform X1	[R].IHGSAAREEDEHPYELLLTAETK.[K]	Y476	1	0.169672701	0.048986149
XP_016875215.1	PXN	5829	paxillin isoform X1	[R].VGEEEHVYSFPNK.[Q]	Y124	230	0.53931046	0.022614079
XP_016875215.1	PXN	5829	paxillin isoform X1	[R].VGEEEHVYSFPNKQK.[S]		119	1.020260635	0.912192794
XP_006721930.1	ITGB4	3691	integrin beta-4 isoform X3	[K].VCAYGAQGEGPYSSLVSCR.[T]	Y1242	75	0.652133927	0.087204052
XP_011536034.1	CDK2	1017	cyclin-dependent kinase 2 isoform X1	[K].VEKIGEGTYGVVYK.[A]		14	42.3579488	0.077738816
NP_001292553.1	CALM2	805	calmodulin-2 isoform 1	[R].VFDKDGNGYISAAELR.[H]	Y148	43	2.559473347	0.008853406
NP_002148.1	HSPE1	3336	10 kDa heat shock protein, mitochondrial	[K].VLLPEYGGTK.[V]	Y76	16	0.635487336	0.066203535
NP_003932.3	WASL	8976	neural Wiskott-Aldrich syndrome protein	[K].VIYDFIEK.[T]	Y256	45	0.868933012	0.223413959
NP_058642.1	F11R	50848	junctional adhesion molecule A isoform 1 precursor	[K].VIYSQPSAR.[S]	Y280	18	0.720871821	0.376217692
NP_001291465.1	EPHA4	2043	ephrin type-A receptor 4 isoform a precursor	[R].VLEDDPEAAYTTR.[G]	T780	87	0.532750513	0.008967604
NP_004422.2	EPHA2	1969	ephrin type-A receptor 2 isoform 1 precursor	[R].VLEDDPEATYTTSGGK.[I]	Y772	70	0.400183598	0.008112786
NP_004422.2	EPHA2	1969	ephrin type-A receptor 2 isoform 1 precursor	[R].VLEDDPEATYTTSGGKIPIR.[W]		279	1.02792507	0.818744924
NP_002341.1	LYN	4067	tyrosine-protein kinase Lyn isoform A	[R].VIEDNEYTAR.[E]	Y397	46	0.444753547	0.020066169
NP_002341.1	LYN	4067	tyrosine-protein kinase Lyn isoform A	[R].VIEDNEYTAREGAK.[F]	Y397	28	0.828658375	0.572688935
NP_001291465.1	EPHA4	2043	ephrin type-A receptor 4 isoform a precursor	[R].TYVDPFTYEDPNQAVR.[E]		49	0.729592106	0.563962404
NP_004422.2	EPHA2	1969	ephrin type-A receptor 2 isoform 1 precursor	[K].TYVDPHTYEDPNQAVLK.[F]		115	0.653099317	0.011075807
XP_011510318.1	PKP4	8502	plakophilin-4 isoform X1	[R].TYYSPVYR.[S]		11	0.445849169	0.092185523
NP_002736.3	MAPK1	5594	mitogen-activated protein kinase 1	[R].VADPDHDHTGFLTEYVATR.[W]	Y187	186	1.147463485	0.457127229
NP_002736.3	MAPK1	5594	mitogen-activated protein kinase 1	[R].VADPDHDHTGFLTEYVATR.[W]	T185	4	1.38027784	0.396482212
NP_004422.2	EPHA2	1969	ephrin type-A receptor 2 isoform 1 precursor	[K].TYVDPHTYEDPNQAVLK.[F]	Y594	32	0.319590652	0.001120434
NP_001278910.1	PTPRK	5796	receptor-type tyrosine-protein phosphatase kappa isoform c precursor	[R].YLCEGTESPYQTGQLHPAIR.[V]	Y875	2	0	0.142239111
NP_004094.3	PTK2B	2185	protein-tyrosine kinase 2-beta isoform a	[R].YIEDEDYYKASVTRLPIK.[W]	Y579; Y580	16	4.612403452	0.040287341
NP_004406.2	DSP	1832	desmoplakin isoform I	[K].YGDGIQLTR.[S]	Y95	6	0.424107627	0.067914027
NP_001008709.1	PPP1CA	5499	serine/threonine-protein phosphatase PP1-alpha catalytic subunit isoform 3	[K].YGQFSGLNPGGRPITPPR.[N]	Y317	28	1.201051515	0.286673419
NP_004330.1	PTTG1IP	754	pituitary tumor-transforming gene 1 protein-interacting protein isoform 1 precursor	[K].YGLFKEENPYAR.[F]	Y174	73	2.136855255	0.009222151

Najar et al; Supplementary Table S1: List of phosphopeptides identified and quantified in LFQ based quantitative phosphoproteomic approach in gastric cancer cells (AGS) upon CAMKK2 inhibition.

NP_001339626.1	PTK2	5747	focal adhesion kinase 1 isoform e;ref|XP_024302967.1	[R].YMEDSTYYK.[A]	Y662	92	0.220160882	0.000222723
NP_067018.2	CLDN6	9074	claudin-6 precursor	[R].YSTSAPAISR.[G]	Y/S	40	1.871041612	0.012177069
NP_001191215.1	MUC1	4582	mucin-1 isoform 10 precursor	[R].YVPPSSTDRSPYEK.[V]	Y458	111	0.636137553	0.018096449
NP_002529.1	OCLN	100506658	occludin isoform a	[R].YSSGGNFETPSK.[R]		27	0.928635356	0.747206469
NP_001491.1	GMDS	2762	GDP-mannose 4,6 dehydratase isoform 1	[K].YYRPTEVDFLQGDCTK.[A]		69	0.834605864	0.091778487
NP_001339626.1	PTK2	5747	focal adhesion kinase 1 isoform e;ref|XP_024302967.1	[R].YMEDSTYYK.[A]	Y661	18	0.055435321	0.000478846
NP_001339626.1	PTK2	5747	focal adhesion kinase 1 isoform e;ref|XP_024302967.1	[R].YMEDSTYYKASK.[G]		39	0.419498039	0.04582326
NP_001339626.1	PTK2	5747	focal adhesion kinase 1 isoform e;ref|XP_024302967.1	[R].YMEDSTYYKASK.[G]	Y661; Y662	37	0.189170898	0.003863746
XP_024305828.1	PEAK1	79834	pseudopodium-enriched atypical kinase 1 isoform X1	[R].YQEVWTSSTSPR.[Q]	Y531	32	2.165745497	0.016909131
XP_016881449.1	YES1	7525	tyrosine-protein kinase Yes isoform X1	[K].YRPENTPEPVSTSVSHYGAEPTTVSPCPSSSAK.[G]		31	0.21507229	0.134527824
XP_016882334.1	NUCB1	4924	nucleobindin-1 isoform X1	[R].YEMLKEHER.[R]	Y179	6	0.048975394	0.003169717
XP_024306664.1	STAT3	6774	signal transducer and activator of transcription 3 isoform X5	[K].YCRPESQEHPEADPGSAAPYLK.[T]	Y737	137	0.348007411	0.000297253
NP_002827.1	PTPRA	5786	receptor-type tyrosine-protein phosphatase alpha isoform 1 precursor	[K].VVQEYIDAFSDYANFK.[-]	Y/S	9	4.137254602	0.19171438
XP_005257569.1	GPRC5C	55890	G-protein coupled receptor family C group 5 member C isoform X1	[K].VPSEGAYDIILPR.[A]	Y487	47	1.10811009	0.505664287
NP_000393.4	G6PD	2539	glucose-6-phosphate 1-dehydrogenase isoform a	[R].VQPNEAVYTK.[M]	Y431	14	0.618085271	0.121142374
NP_001317366.1	PTPN11	5781	tyrosine-protein phosphatase non-receptor type 11 isoform 3	[R].VYENVGLMQQQK.[S]	Y584	9	0	0.043900581
XP_016860997.1	TNK2	10188	activated CDC42 kinase 1 isoform X3	[K].VSSTHYYLLPERPSYLER.[Y]	Y891	61	1.209238764	0.653105416
NP_036441.2	SHANK2	22941	SH3 and multiple ankyrin repeat domains protein 2 isoform 1	[R].VYGTIKPAFNQNSAAK.[V]	Y917	4	0.573653986	0.605225653
XP_016881449.1	YES1	7525	tyrosine-protein kinase Yes isoform X1	[K].WTAPEAALYGR.[F]	Y446	30	0.767093024	0.081682164
NP_005611.1	S100A11	6282	protein S100-A11	[K].YAGKDGYNYTLSK.[T]	Y32	13	0.749393508	0.489429039
XP_016860997.1	TNK2	10188	activated CDC42 kinase 1 isoform X3	[K].YATPQVIQAPGPR.[A]	T861	75	0.863932446	0.308790609
XP_005257673.2	STAT3	6774	signal transducer and activator of transcription 3 isoform X2	[K].YCRPESQEHPEADPGAAPYLK.[T]	Y705	76	0.296394331	0.011822462
XP_011541606.1	SERINC5	256987	serine incorporator 5 isoform X1	[R].YAAPELEIAR.[C]	Y345	20	0.755949197	0.09124557
NP_006473.2	DYRK2	8445	dual specificity tyrosine-phosphorylation-regulated kinase 2 isoform 2	[RK].VYTYIQSR.[F]	Y382	74	0.81127587	0.261288029
NP_001135865.1	NEDD9	4739	enhancer of filamentation 1 isoform 3	[R].TGHGYVYEYPSR.[Y]	Y166	51	0.902439828	0.754576985
NP_004437.2	EPRS	2058	bifunctional glutamate/proline--tRNA ligase	[K].TGKEYIPGQPPLSQSSDSSPTR.[N]	S886	17	2.574177019	0.086313956
NP_004437.2	EPRS	2058	bifunctional glutamate/proline--tRNA ligase	[K].TGKEYIPGQPPLSQSSDSSPTRNSEPAGLETPEAK.[V]		98	8.256342983	0.006429635
NP_061918.3	WDR44	54521	WD repeat-containing protein 44 isoform 1	[R].TKEYVSNDAAQSDDEEKLQSQPTDTDGGR.[L]		11	9.278655559	0.425149279
XP_011513536.1	COBL	23242	protein cordon-bleu isoform X1	[R].TSSQYVASAIAK.[R]	Y964	9	1.675097629	0.049433572
XP_005252786.1	HIPK3	10114	homeodomain-interacting protein kinase 3 isoform X1	[K].TVCSTYLQSR.[Y]	Y359	32	0.66797263	0.061169947
NP_001092.1	ACTB	60	actin, cytoplasmic 1	[R].GYSFTTTAER.[E]	Y198	13	0.676921821	0.356616255
XP_024302967.1	PTK2	5747	focal adhesion kinase 1 isoform X1	[R].GSIDREDGSLQGPIGNQHIYQPVGKPDPAAPPK.[K]	Y925	45	0.290782159	0.029393221
XP_011537724.1	DOCK1	1793	dedicator of cytokinesis protein 1 isoform X1	[K].GSVADYGNLMENQDLLGSPTPPPPPPHQR.[H]	Y/S/T	10	0	0.116176596
XP_016860272.1	STAT1	6772	signal transducer and activator of transcription 1-alpha/beta isoform X1	[K].GTGYIKTELISVSEVHPSR.[L]		47	1.540583436	0.263572763
NP_002745.1	MAPK13	5603	mitogen-activated protein kinase 13	[R].HADAEMTGYVVTR.[W]	Y182	153	1.160334642	0.404292367
NP_001171669.1	CTTN	2017	src substrate cortactin isoform c;ref|NP_005222.2	[K].HASQKDYSSGFGGK.[Y]		19	0.571804385	0.165820242
NP_056474.2	RSL1D1	26156	ribosomal L1 domain-containing protein 1	[K].HATGKKSPAKSPNPSTPR.[G]	S392	18	1.995593806	0.119295483
NP_002745.1	MAPK13	5603	mitogen-activated protein kinase 13	[R].HADAEMTGYVVTR.[W]	Y182	22	1.609153169	0.323478551
NP_005498.1	CFL1	1072	cofilin-1	[K].HELQANCYEEVKDR.[C]	Y140	6	0.095580369	0.019422035
NP_001289958.1	PKP3	11187	plakophilin-3 isoform PKP3b	[R].GQYHTLQAGFSSR.[S]	T101	21	0.985807755	0.966242847
XP_005245072.1	PRRC2C	23215	protein PRRC2C isoform X1	[R].GHTRDYPQYR.[D]	Y1220	74	1.093589916	0.681763584
NP_067018.2	CLDN6	9074	claudin-6 precursor	[R].GPSEYPTKNYV.[-]	Y219	28	0.434697242	0.093904351
XP_024304270.1	INPPL1	3636	phosphatidylinositol 3,4,5-trisphosphate 5-phosphatase 2 isoform X1	[R].GLPSDYGRPLSFPPPR.[I]	Y1228	20	0.426253378	0.002165048
NP_001008709.1	PPP1CA	5499	serine/threonine-protein phosphatase PP1-alpha catalytic subunit isoform 3	[K].GKYGQFSGLNPGGRPITPPR.[N]	Y317	2	#DIV/0!	#DIV/0!
XP_006721622.1	MAPK7	5598	mitogen-activated protein kinase 7 isoform X1	[R].GLCTSPAEHQYFMTEYVATR.[W]	T219;Y221	9	0.068205195	0.029105919

Najar et al; Supplementary Table S1: List of phosphopeptides identified and quantified in LFQ based quantitative phosphoproteomic approach in gastric cancer cells (AGS) upon CAMKK2 inhibition.

XP_005266937.1	FRK	2444	tyrosine-protein kinase FRK isoform X1	[R].HGHYFVALFDYQAR.[T]	Y46	143	1.784354645	0.220846353
NP_001135865.1	NEDD9	4739	enhancer of filamentation 1 isoform 3	[R].HQSLSPNHPPPQLGQSVGSQNDAYDVPR.[G]	Y317	11	0.923311638	0.922214889
XP_011512612.2	MAPK14	1432	mitogen-activated protein kinase 14 isoform X1	[R].HTDDEMTGYVATR.[W]	Y223	88	0.650601625	0.017607105
XP_011512612.2	MAPK14	1432	mitogen-activated protein kinase 14 isoform X1	[R].HTDDEMTGYVATR.[W]	Y223	33	0.813308204	0.729634257
NP_542390.2	C11orf52	91894	uncharacterized protein C11orf52	[K].HVHLENATEYATLR.[F]		100	1.130309261	0.411727768
NP_542390.2	C11orf52	91894	uncharacterized protein C11orf52	[K].HVHLENATEYATLRFPQATPR.[Y]	Y/T	7	11.9728183	0.013434671
NP_001157918.1	C6orf132	647024	uncharacterized protein C6orf132	[K].HKAPGSADYGFAPAAGR.[S]	Y1142	2	0.701399244	0.578855185
XP_011518907.1	EPS8	2059	epidermal growth factor receptor kinase substrate 8 isoform X1	[R].HIDRNYEPLKTQPK.[K]	Y545	7	1.151247417	0.772218866
NP_001171725.1	ESYT1	23344	extended synaptotagmin-1 isoform 1	[K].HLSPYATLTVGDSSHK.[T]	Y/S	23	0.868015944	0.441286604
NP_002861.1	RAB13	5872	ras-related protein Rab-13 isoform 1	[K].AYDHLFK.[L]	Y5	6	1.145870957	0.718631322
NP_001002857.1	ANXA2	302	annexin A2 isoform 2	[K].AYTNFDAER.[D]	Y30	24	0.975002372	0.94388817
XP_011514379.1	HIPK2	28996	homeodomain-interacting protein kinase 2 isoform X1	[K].AVCSTYLQSR.[Y]	Y540	181	0.912924628	0.408599599
XP_016865124.1	ERBIN	55914	erbin isoform X1	[R].AQIPEGDYLSYR.[E]	Y1104	25	0.355886959	0.002286539
NP_001164185.1	BCAR1	9564	breast cancer anti-estrogen resistance protein 1 isoform 1	[K].AQQGLYQVPGPSPQFQSPPAK.[Q]	Y174	52	1.932085037	0.059492405
NP_001018146.1	NME1-NME2	654364	NME1-NME2 protein	[R].ASEEHLKQHYIDLK.[D]	Y167	9	0.824749733	0.369852947
NP_003019.2	SHB	6461	SH2 domain-containing adapter protein B	[K].DKVTIADDYSDPFDAKNDLK.[S]		25	0.609918043	0.340510651
NP_001135865.1	NEDD9	4739	enhancer of filamentation 1 isoform 3	[R].DEAGLREKDYDFPPPMR.[Q]	Y241	80	0.308682234	0.032700332
NP_001302.1	CRIP1	1396	cysteine-rich protein 1	[K].CNKEVYFAER.[V]	Y12	36	0.730563697	0.342021113
NP_003019.2	SHB	6461	SH2 domain-containing adapter protein B	[K].AGKGESAGYMEPYEAQR.[I]	Y268	44	0.37460284	0.016037319
NP_003970.1	GPRC5A	9052	retinoic acid-induced protein 3	[R].AHAWPSPYKDYEVKK.[E]	Y347	34	0.869493798	0.555406625
XP_005266998.1	DCBLD1	285761	discoidin, CUB and LCCL domain-containing protein 1 isoform X1	[R].AHTFSAQSGYRVPGPQPGHK.[H]	Y668	38	1.556007925	0.432111598
NP_004563.2	PKP2	5318	plakophilin-2 isoform 2b	[R].AHYTHSDYQYSQR.[S]	S164	40	0.552462281	0.069389056
NP_001289958.1	PKP3	11187	plakophilin-3 isoform PKP3b	[R].ADYDTLSLR.[S]	Y191	14	0.367496641	0.016353159
NP_004563.2	PKP2	5318	plakophilin-2 isoform 2b	[M].AAPGAPAEYGYIR.[T]	Y10	44	0.817614978	0.196501019
NP_991403.1	LSR	51599	lipolysis-stimulated lipoprotein receptor isoform 2	[K].AATSGVPSIYAPSTYAHLSPAK.[T]	Y328	95	1.358308085	0.04449477
NP_758869.1	CD46	4179	membrane cofactor protein isoform 2 precursor	[K].ADGGAEYATYQTK.[S]	Y387	93	0.41314349	0.014894054
NP_001135865.1	NEDD9	4739	enhancer of filamentation 1 isoform 3	[K].ANPQERDGVYDVPLHNPPDAK.[G]	Y345	18	0.643226778	0.256536597
XP_005257069.1	CLTC	1213	clathrin heavy chain 1 isoform X1	[R].ALEHFTDLYDIKR.[A]	Y634	16	1.052858722	0.663707157
XP_016867305.1	EPHB4	2050	ephrin type-B receptor 4 isoform X1	[R].FLEENSSDPTYTSSLGGK.[I]	Y/T	6	0.329869095	0.004196048
XP_016867305.1	EPHB4	2050	ephrin type-B receptor 4 isoform X1	[R].FLEENSSDPTYTSSLGGKIPIR.[W]	T/Y	5	#DIV/0!	#DIV/0!
XP_016875215.1	PXN	5829	paxillin isoform X1	[R].FIHQQPQSSSPVYGSSAK.[T]	Y94	208	0.868825831	0.248748571
NP_001339626.1	PTK2	5747	focal adhesion kinase 1 isoform e	[R].FLIGNQHIYQPVGKPDPAAPPK.[K]	Y925	24	0.486951487	0.006261617
XP_016875215.1	PXN	5829	paxillin isoform X1	[R].FIHQQPQSSSPVYGSSAK.[T]	S91; Y94	88	0.590592235	0.054773362
NP_063937.2	GSK3A	2931	glycogen synthase kinase-3 alpha	[R].GEPNVSYICSR.[Y]	Y279	684	0.972264117	0.89378767
NP_003019.2	SHB	6461	SH2 domain-containing adapter protein B	[K].GESAGYMEPYEAQR.[I]		22	0	0.019565427
XP_016878010.1	TJP1	7082	tight junction protein ZO-1 isoform X1	[R].FTPKPYTSSARPFER.[K]	Y1642	15	2.553380154	0.041187777
NP_001393.1	EEF1A1	1915	elongation factor 1-alpha 1	[R].EHALLAYTLGVK.[Q]	Y/T	34	1.004855117	0.978724389
XP_005245506.1	SHC1	6464	SHC-transforming protein 1 isoform X1	[R].ELFDDPSYVNVQNLDK.[A]	Y/S	33	1.074627886	0.92631768
NP_062565.2	PARD3	56288	partitioning defective 3 homolog isoform 1	[R].ERDYAEIQDFHR.[T]	Y1080	18	0.45068751	0.012757575
XP_024304270.1	INPPL1	3636	phosphatidylinositol 3,4,5-trisphosphate 5-phosphatase 2 isoform X1	[R].ERLYEWISIDKDEAGAK.[S]	Y952	10	0.637658046	0.070405929
XP_016881449.1	YES1	7525	tyrosine-protein kinase Yes isoform X1	[R].ESETTKGAYSLSIR.[D]	S195	14	0.595264992	0.144611112
NP_003932.3	WASL	8976	neural Wiskott-Aldrich syndrome protein	[R].ETSKVIYDFIEK.[T]	Y256	21	1.274208558	0.130899973
NP_003080.2	SNRNP70	6625	U1 small nuclear ribonucleoprotein 70 kDa isoform 1	[R].EFEVYGPIKR.[I]	Y126	74	1.535853614	0.019433815
XP_016867305.1	EPHB4	2050	ephrin type-B receptor 4 isoform X1	[R].EAEYSDKHGQYLIGHGTK.[V]	Y/S	73	0.470281003	0.027253596
XP_016867305.1	EPHB4	2050	ephrin type-B receptor 4 isoform X1	[R].EAEYSDKHGQYLIGHGTK.[V]	Y592; Y599	7	1.107696893	0.77105686
NP_001074295.2	PRAG1	157285	inactive tyrosine-protein kinase PRAG1	[R].EATQPEPIYAESTK.[R]	T417	42	1.190432797	0.601206138
NP_001074295.2	PRAG1	157285	inactive tyrosine-protein kinase PRAG1	[R].EATQPEPIYAESTKR.[K]	Y413	163	1.590514394	0.098402778
XP_006721930.1	ITGB4	3691	integrin beta-4 isoform X3	[R].DYSTLTSVSSHDSR.[L]	Y1545	22	0.095844807	0.003723762

Najar et al; Supplementary Table S1: List of phosphopeptides identified and quantified in LFQ based quantitative phosphoproteomic approach in gastric cancer cells (AGS) upon CAMKK2 inhibition.

NP_001393.1	EEF1A1	1915	elongation factor 1-alpha 1	[K].FETSKYYVTIIDAPGHR.[D]		74	0.8368205	0.562747247
NP_002957.1	S100A10	6281	protein S100-A10	[K].FAGDKGYLTKEDLR.[V]	Y25	30	1.590029261	0.016976126
NP_006808.1	ERP29	10961	endoplasmic reticulum resident protein 29 isoform 1 precursor	[K].FDTQYPYGEKQDEFKR.[L]	Y66	96	1.40126641	0.088540598
NP_001078927.1	CTNND1	1500	catenin delta-1 isoform 1ABC	[R].HYEDGYPGGSDNYGSLSR.[V]	Y228	25	0.239437199	0.001509234
XP_011513778.1	TNS3	64759	tensin-3 isoform X2	[R].KLSLGQYDNDAGGQLPFSK.[C]	Y896	48	1.042055186	0.833332497
NP_001189399.1	RPS10-NUDT3	100529239	RPS10-NUDT3 protein	[R].IAIYELLFK.[E]	Y12	4	4.882969658	0.381754324
NP_001307847.1	CDK1	983	cyclin-dependent kinase 1 isoform 1;ref|XP_011536034.1	[K].IGEGTYGVVYK.[AG]	T14; Y15	240	1.910186373	0.052542586
NP_001307847.1	CDK1	983	cyclin-dependent kinase 1 isoform 1	[K].IGEGTYGVVYKGR.[H]	Y19	26	1.138087489	0.672947908
NP_001307847.1	CDK1	983	cyclin-dependent kinase 1 isoform 1	[K].IGEGTYGVVYKGR.[H]	T14; Y15	111	9.216330941	0.0353332
NP_002793.2	PSMC1	5700	26S proteasome regulatory subunit 4 isoform a	[K].KKYEPPVPTR.[V]	Y25	23	1.007633203	0.983683279
NP_004926.1	CDK5	1020	cyclin-dependent-like kinase 5 isoform 1	[K].IGEGTYGTVFK.[A]		94	1.672801989	0.046357817
NP_001307847.1	CDK1	983	cyclin-dependent kinase 1 isoform 1;ref|XP_011536034.1	[K].IGEGTYGVVYK.[AG]		600	0.998200836	0.992232681
XP_016881449.1	YES1	7525	tyrosine-protein kinase Yes isoform X1	[R].KLDNGGYYITTR.[A]	Y223	30	1.378199634	0.444371082
XP_016860997.1	TNK2	10188	activated CDC42 kinase 1 isoform X3	[K].KVSSTHYYLLPERPSYLER.[Y]	Y891	9	0.84708264	0.540008449
NP_058642.1	F11R	50848	junctional adhesion molecule A isoform 1 precursor	[K].KVIYSQPSAR.[S]	Y280	21	0.929800211	0.848076618
NP_001372.1	DOK1	1796	docking protein 1 isoform a	[R].IAPCPSQDSLYSDPLDSTSAQAGEGVQR.[K]		66	0.618864847	0.361815048
NP_002737.2	MAPK3	5595	mitogen-activated protein kinase 3 isoform 1	[R].IADPEHDHTGFLTEYVATR.[W]	Y204	144	1.491273334	0.040976966
NP_004330.1	PTTG1IP	754	pituitary tumor-transforming gene 1 protein-interacting protein isoform 1 precursor	[K].KYGLFKEENPYAR.[F]	Y174	16	1.555153804	0.453786593
NP_001337261.1	TOM1L2	146691	TOM1-like protein 2 isoform 9	[R].KTVTYEDPQAVGGLASALDNRK.[Q]		46	0.918078961	0.742121644
NP_003019.2	SHB	6461	SH2 domain-containing adapter protein B	[R].LDYCGGSGEPGGVQR.[A]	Y114	34	0.466872818	0.069698548
NP_001307847.1	CDK1	983	cyclin-dependent kinase 1 isoform 1	[K].IEKIGEGTYGVVYK.[G]	T14	60	7.745508969	0.037039042
NP_001307847.1	CDK1	983	cyclin-dependent kinase 1 isoform 1	[K].IEKIGEGTYGVVYK.[G]	T14; Y15	94	5.559817975	0.013827571
NP_001163887.1	TJP2	9414	tight junction protein ZO-2 isoform 3	[R].IEIAQKHPDIYAVPIK.[T]	Y1149	32	1.262890915	0.198458073
XP_016860997.1	TNK2	10188	activated CDC42 kinase 1 isoform X3	[K].KPTYDPVSEDQDPLSSDFKR.[L]	T/Y	86	1.215009198	0.305378495
NP_001347.3	DDX3X	1654	ATP-dependent RNA helicase DDX3X isoform 1	[R].KQYPISLVLAPTR.[E]	Y266	6	0.785646488	0.349158571
XP_016866912.1	PRPF4B	8899	serine/threonine-protein kinase PRP4 homolog isoform X1	[K].LCDFGSASHVADNDITPYLVSR.[F]	Y849	790	0.642015471	0.525589405
NP_001337261.1	TOM1L2	146691	TOM1-like protein 2 isoform 9	[R].KTVTYEDPQAVGGLASALDNR.[K]		32	0.563922917	0.336042787
XP_005260495.1	PLCG1	5335	1-phosphatidylinositol 4,5-bisphosphate phosphodiesterase gamma-1 isoform X1	[K].IGTAEPDYGALYEGR.[N]	Y771	11	0.89245008	0.574444787
NP_002620.1	PGAM1	5223	phosphoglycerate mutase 1 isoform 1	[R].HYGGLTGLNKAETAAK.[H]	Y92	16	1.715078186	0.126770761
NP_002620.1	PGAM1	5223	phosphoglycerate mutase 1 isoform 1	[R].HYGGLTGLNK.[A]	Y92	30	0.583901372	0.065815222
XP_024302117.1	EPB41L2	2037	band 4.1-like protein 2 isoform X7	[K].QKSYTLVVAK.[D]	S87	11	0.852794333	0.485725744
NP_001289958.1	PKP3	11187	plakophilin-3 isoform PKP3b	[R].GGVGSRADYDTLSLR.[S]		8	1.072883123	0.810259204
NP_006334.2	MERTK	10461	tyrosine-protein kinase Mer precursor	[RK].KIYSGDYYR.[Q]	Y749	15	0.722825516	0.339193945
XP_016865124.1	ERBIN	55914	erbin isoform X1	[R].RAQIPEGDYLSYR.[E]	Y1104	10	0.309545944	0.022053723
NP_005966.1	PTK6	5753	protein-tyrosine kinase 6 isoform 1	[R].VSEKPSADYVLSVR.[D]		3	1.284406891	0.212814861
NP_000692.2	ATP1A1	476	sodium/potassium-transporting ATPase subunit alpha-1 isoform a	[R].GIVVYTGDR.[T]	Y260	24	0.465869389	0.027971323
NP_001341605.1	PRKCD	5580	protein kinase C delta type isoform a	[K].GRGEYFAIK.[A]	Y393	12	0.98370566	0.954644441
XP_005257141.1	WIPF2	147179	WAS/WASL-interacting protein family member 2 isoform X1	[K].GSSGGYGSGGAALQPK.[G]	S70	21	0.446658335	0.025394761
NP_003322.3	TYK2	7297	non-receptor tyrosine-protein kinase TYK2	[R].LLAQAEGEPCYIR.[D]	Y292	4	0.643215995	0.375998317
NP_005223.4	EPHA1	2041	ephrin type-A receptor 1 precursor	[R].LLDDFDGTYETQGGK.[I]	Y781	7	0.366373526	0.080659032
NP_054706.1	VCL	7414	vinculin isoform meta-VCL	[K].SFLDSGYRILGAVAK.[V]	S820	10	5.77813506	0.225187544
XP_005266998.1	DCBLD1	285761	discoidin, CUB and LCCL domain-containing protein 1 isoform X1	[K].AVSALATESGHPDSQKPPTHPGTSDSYSAPR.[D]		31	1.468035831	0.737224661
NP_001329.1	CXADR	1525	coxsackievirus and adenovirus receptor isoform 1 precursor	[K].TQYNQVPSEDFER.[T]	Y318	15	0.321933894	0.006985535
NP_001333375.1	AHNAK	79026	neuroblast differentiation-associated protein AHNAK isoform 1	[K].VKGEYDVTVPKLEGELKGPK.[V]	Y715	8	0.305924979	0.023065486
NP_001333375.1	AHNAK	79026	neuroblast differentiation-associated protein AHNAK isoform 1	[K].VKGEYDVTVPK.[L]	Y715	18	0.184323133	0.001049076

Najar et al; Supplementary Table S1: List of phosphopeptides identified and quantified in LFQ based quantitative phosphoproteomic approach in gastric cancer cells (AGS) upon CAMKK2 inhibition.

XP_024308698.1	ITSN2	50618	intersectin-2 isoform X1	[K].REEPEALYAAVNK.[K]	Y968	8	0.118142692	0.000297848
NP_006808.1	ERP29	10961	endoplasmic reticulum resident protein 29 isoform 1 precursor	[K].FDTQYPYGEKQDEFK.[R]	Y66	8	0.313701654	0.082945984
XP_016869431.1	ADAM9	8754	disintegrin and metalloproteinase domain-containing protein 9 isoform X1	[R].HVSPVTPPREVPIYANR.[F]	Y771	3	#DIV/0!	0.180473262
XP_016869431.1	ADAM9	8754	disintegrin and metalloproteinase domain-containing protein 9 isoform X1	[R].HVSPVTPPREVPIYANR.[F]	T763; Y771	29	1.313095249	0.054005725
NP_001164185.1	BCAR1	9564	breast cancer anti-estrogen resistance protein 1 isoform 1	[R].VLPPEVADGGVVDSGVYAVPPPAER.[E]	Y456	7	0.431003431	0.098057857
XP_005262892.1	FAT1	2195	protocadherin Fat 1 isoform X1	[K].KPLEEKPSQPYSAR.[E]	S4357	23	0.071096889	0.003305219
XP_005266998.1	DCBLD1	285761	discoidin, CUB and LCCL domain-containing protein 1 isoform X1	[K].HSLSSGGFSPVAGVGAQDGDYQRPHSAQPADRGYDRPK.[A]		5	#DIV/0!	#DIV/0!
NP_002620.1	PGAM1	5223	phosphoglycerate mutase 1 isoform 1	[R].FSGWYDADLSPAGHEEAKR.[G]	Y26	4	#DIV/0!	#DIV/0!
XP_016865074.1	PIK3R1	5295	phosphatidylinositol 3-kinase regulatory subunit alpha isoform X1	[KR].EYDRLYEEYTR.[T]	Y463	2	1.089916576	0.909149136
NP_001092.1	ACTB	60	actin, cytoplasmic 1	[R].FRCPEALFQPSFLGMESCGIHETTFNSIMK.[C]		2	#DIV/0!	#DIV/0!
NP_055843.1	TNIK	23043	TRAF2 and NCK-interacting protein kinase isoform 1	[R].DYLVSLQHQR.[Q]	Y499	5	1.378017068	0.519926942
NP_006280.3	TLN1	7094	talin-1	[K].ALDYYMLR.[N]	Y70	26	0.610046107	0.479974957
NP_002939.2	RPL15	6138	60S ribosomal protein L15 isoform 1	[K].AKQGYVIYR.[I]	Y59	17	1.279425424	0.521848906
XP_011518907.1	EPS8	2059	epidermal growth factor receptor kinase substrate 8 isoform X1	[R].HIDRNYEPLK.[T]	Y545	11	0.401588027	0.005973283
NP_001336850.1	RPL27	6155	60S ribosomal protein L27	[K].NIDDGTSDRPYSHALVAGIDRYPR.[K]	Y49	2	0.835479817	0.704270033
NP_003970.1	GPRC5A	9052	retinoic acid-induced protein 3	[R].AHAWPSPYKDYEVKKEGS.[-]	Y350	25	1.996515612	0.093299114
NP_061849.2	SLC38A2	54407	sodium-coupled neutral amino acid transporter 2 isoform 1	[K].SHYADVDPENQNFLLESNLGK.[K]	Y41	3	#DIV/0!	#DIV/0!
NP_003970.1	GPRC5A	9052	retinoic acid-induced protein 3	[R].AHAWPSPYKDYEVKK.[E]	Y347; Y350	13	0.475105292	0.115690803
NP_001163887.1	TJP2	9414	tight junction protein ZO-2 isoform 3	[K].HPDIYAVPIK.[T]	Y1149	8	0.564142718	0.381454043
NP_003970.1	GPRC5A	9052	retinoic acid-induced protein 3	[R].AHAWPSPYKDYEVKKEGS.[-]	S345; Y350	41	1.219496467	0.598063167
NP_001164185.1	BCAR1	9564	breast cancer anti-estrogen resistance protein 1 isoform 1	[R].HLLAPGPQDIYDVPPVR.[G]	Y295	15	0.987387023	0.979279857
NP_004094.3	PTK2B	2185	protein-tyrosine kinase 2-beta isoform a	[R].YIEDEDYYKASVTR.[L]	Y579; Y580	5	14.14488411	0.145803213
XP_005245231.2	MGST3	4259	microsomal glutathione S-transferase 3 isoform X1	[K].YKVEYPIMYSTDPENGHIFNCIQR.[A]	Y40	5	2.042377837	0.578952077
XP_005251926.1	CEMIP2	23670	cell surface hyaluronidase isoform X1	[R].HPSGYVPGK.[V]	Y27	4	0.107485024	0.0024977
NP_002939.2	RPL15	6138	60S ribosomal protein L15 isoform 1	[K].QGYVIYR.[I]	Y59	4	0.711726081	0.257924172
XP_016875895.1	EFNB2	1948	ephrin-B2 isoform X1	[R].TADSVFCPHYEK.[V]	Y306	21	0.157934278	0.002551283
NP_002737.2	MAPK3	5595	mitogen-activated protein kinase 3 isoform 1	[R].IADPEHDHTGFLTEYVATR.[W]	T202; Y204	2	12.75319273	0.026983662
XP_005257569.1	GPRC5C	55890	G-protein coupled receptor family C group 5 member C isoform X1	[R].GVGYETILK.[E]	Y424	11	0.526758204	0.047151145
NP_596867.1	ITGB1	3688	integrin beta-1 isoform 1A precursor	[K].WDTGENPIYK.[S]	Y783	4	0.626013131	0.653771152
XP_016861292.1	DLG1	1739	disks large homolog 1 isoform X1	[K].RDYEVDGRDYHFVTSR.[E]	Y782	1	0.911111254	0.88575788
NP_001527.3	PRMT1	3276	protein arginine N-methyltransferase 1 isoform 1	[R].TGFSTSPESPYTHWK.[Q]	Y309	8	0.416176145	0.006331676
NP_001334757.1	PDGFRA	5156	platelet-derived growth factor receptor alpha isoform 3	[R].SLYDRPASYK.[K]	Y787; Y793	2	0	0.116625223
XP_011530744.1	LOC105377310	105377310	syncytin-1-like	[R].VPILPFVTGAGVLGR.[L]	T29	26	1.220238057	0.790924893
XP_024305828.1	PEAK1	79834	pseudopodium-enriched atypical kinase 1 isoform X1	[K].VPIVINPNAYDNLAIYK.[S]	Y635	15	0.241817946	0.314348056
NP_991403.1	LSR	51599	lipolysis-stimulated lipoprotein receptor isoform 2	[R].VLYYMEK.[E]	Y406	8	0.505400828	0.021392899
NP_001121620.1	TFRC	7037	transferrin receptor protein 1 isoform 1	[R].SAFSNLFGGEPLSYTR.[F]	Y20	9	0.847272322	0.828067688
NP_001393.1	EEF1A1	1915	elongation factor 1-alpha 1	[K].YYVTIIDAPGHR.[D]	T88	3	0.182434103	0.057640042
XP_016881449.1	YES1	7525	tyrosine-protein kinase Yes isoform X1	[K].GAYSLSIRDWDEIRGDNVK.[H]	Y194	3	0.495876766	0.27957374
XP_016881449.1	YES1	7525	tyrosine-protein kinase Yes isoform X1	[K].GAYSLSIR.[D]	Y194	20	0.368997895	0.032641363
NP_001092.1	ACTB	60	actin, cytoplasmic 1	[K].IWHHTFYNELR.[V]	Y91	2	0.697471376	0.163075501
NP_001021.1	RPS27	6232	40S ribosomal protein S27 isoform 1	[R].LVQSPNSYFMDVK.[C]		18	0.564251053	0.084561548
XP_011513778.1	TNS3	64759	tensin-3 isoform X2	[K].ESMCSTPAFPVSPETPYVK.[T]	Y971	6	0.154053617	0.003063068
XP_011542222.1	CDK16	5127	cyclin-dependent kinase 16 isoform X1	[K].LGEGTYATVYK.[G]		7	#DIV/0!	#DIV/0!
NP_003970.1	GPRC5A	9052	retinoic acid-induced protein 3	[R].AHAWPSPYKDYEVK.[K]	Y347	5	0.682276846	0.139173593
XP_016881449.1	YES1	7525	tyrosine-protein kinase Yes isoform X1	[R].ESETTKGAYSLSIRDWDEIRGDNVK.[H]	S/Y	22	3.743218211	0.027910321
XP_005266998.1	DCBLD1	285761	discoidin, CUB and LCCL domain-containing protein 1 isoform X1	[R].AHTFSAQSGYR.[V]	Y668	14	0.73378311	0.639181632
XP_006721930.1	ITGB4	3691	integrin beta-4 isoform X3	[R].SEHSHSTTLPRDYSTLTSVSSHDSR.[L]	Y/T/S	4	1.596132499	0.767452293

Najar et al; Supplementary Table S1: List of phosphopeptides identified and quantified in LFQ based quantitative phosphoproteomic approach in gastric cancer cells (AGS) upon CAMKK2 inhibition.

XP_006710760.1	ERRFI1	54206	ERBB receptor feedback inhibitor 1 isoform X1	[K].VSSTHYYLLPERPPYLDKYEK.[F]	T/S/Y	9	0.08036672	0.233911044
XP_011534403.1	UTRN	7402	utrophin isoform X1	[R].IEQYATR.[L]	Y3233	2	2.276445262	0.316470183
XP_024302967.1	PTK2	5747	focal adhesion kinase 1 isoform X1	[R].FLKPDVRLSRGSIDREDGSLQGPIGNQHIYQPVGKPDPAAPPK.[K]	Y925	15	0	7.85098E-06
XP_016869431.1	ADAM9	8754	disintegrin and metalloproteinase domain-containing protein 9 isoform X1	[R].EVPIYANR.[F]	Y771	27	0.906875004	0.678526353
NP_001066.1	UGT2B10	7365	UDP-glucuronosyltransferase 2B10 isoform 1 precursor	[K].KWDQFYSEVLGRPTTLSETMR.[K]	S246	4	#DIV/0!	#DIV/0!
NP_000692.2	ATP1A1	476	sodium/potassium-transporting ATPase subunit alpha-1 isoform a	[R].KYGTDLSR.[G]	Y55	6	0.07260518	0.001413345
NP_001171669.1	CTTN	2017	src substrate cortactin isoform c;ref|NP_005222.2	[K].NASTFEDVTQVSSAYQK.[T]	Y297	8	0.359256692	0.034182174
NP_054772.1	FLVCR1	28982	feline leukemia virus subgroup C receptor-related protein 1	[K].GYLPLPR.[G]	Y22	14	0.29925566	0.002477274
NP_001347.3	DDX3X	1654	ATP-dependent RNA helicase DDX3X isoform 1	[K].DKDAYSSFGSR.[SD]	Y69	7	0.17496567	0.033793525
NP_000217.2	KRT9	3857	keratin, type I cytoskeletal 9	[R].FSSSSGYGGGSSR.[V]	Y53	2	#DIV/0!	#DIV/0!
NP_002145.3	HSPA4	3308	heat shock 70 kDa protein 4	[K].LKKEDIYAVEIVGGATR.[I]	Y336	5	#DIV/0!	#DIV/0!
NP_006353.2	NXF1	10482	nuclear RNA export factor 1 isoform 1	[R].WKYGEGNRR.[S]	Y33	11	0.171707203	0.000769783
NP_001275647.1	STAT5A	6776	signal transducer and activator of transcription 5A isoform 1	[K].AVDGYVKPQIK.[Q]	Y694	4	1.303768413	0.845012043
XP_024305828.1	PEAK1	79834	pseudopodium-enriched atypical kinase 1 isoform X1	[R].STSSPYHAGNLLQR.[H]	Y880	8	0.170643889	0.02969811
NP_991403.1	LSR	51599	lipolysis-stimulated lipoprotein receptor isoform 2	[R].SRDDLYDQDDSRDFPR.[S]	Y535	2	#DIV/0!	#DIV/0!
NP_002130.2	RBMX	27316	RNA-binding motif protein, X chromosome isoform 1	[R].DYGHSSSRDDYPSR.[G]	Y255	11	0.990370087	0.979873126
NP_001191215.1	MUC1	4582	mucin-1 isoform 10 precursor	[R].DTYHPMSEYPTYHTHGR.[Y]	T/Y	2	#DIV/0!	#DIV/0!
NP_003019.2	SHB	6461	SH2 domain-containing adapter protein B	[K].AGKGESAGYMEPYEAQR.[I]	Y268	6	0	0.060017184
NP_002175.2	IL6ST	3572	interleukin-6 receptor subunit beta isoform 1 precursor	[K].SYLPQTVR.[Q]		4	0.164618355	0.02538177
NP_001372.1	DOK1	1796	docking protein 1 isoform a	[R].VKEEGYELPYNPATDDYAVPPPR.[S]		7	4.655684145	0.339483967
NP_000692.2	ATP1A1	476	sodium/potassium-transporting ATPase subunit alpha-1 isoform a	[K].YGTDLSR.[G]		3	0.169529275	0.04286313
NP_001291465.1	EPHA4	2043	ephrin type-A receptor 4 isoform a precursor	[R].TYVDPFTYEDPNQAVR.[E]		19	0.45787322	0.329750045
NP_054706.1	VCL	7414	vinculin isoform meta-VCL	[K].SFLDSGYR.[I]	Y822	16	0.732051018	0.114745304
NP_835461.2	ZNF598	90850	E3 ubiquitin-protein ligase ZNF598	[R].YKREEEDREVAAAVR.[A]	Y336	2	0.552274342	0.158343537
NP_002130.2	RBMX	27316	RNA-binding motif protein, X chromosome isoform 1	[R].DRDYSDHPSGGSYR.[D]		14	0	0.004949844
NP_077740.1	DSC2	1824	desmocollin-2 isoform Dsc2a preproprotein	[R].YTYSEWHSFTQPR.[L]	T/Y/S	9	0.0732961	0.022650227
NP_001254479.2	TTN	7273	titin isoform IC	[K].VSVGDSASLQCQLAGTPEIGVSWYK.[G]	S9217; S9221; S9223; T9231; S9237; Y9239	1	1.407208831	0.706908107
XP_005268509.2	PCDH1	5097	protocadherin-1 isoform X1	[K].DLYAPKPSGK.[A]	Y913	2	#DIV/0!	#DIV/0!
XP_005264475.1	CCDC88A	55704	girdin isoform X1	[R].RSMSMNDLVQSMVLAGQWTGSTENLEVPDDISTGK.[R]	S1519; T1520	8	2.190050922	0.09341897
NP_006808.1	ERP29	10961	endoplasmic reticulum resident protein 29 isoform 1 precursor	[K].FDTQYPYGEK.[Q]	Y66	2	0	0.241795273
XP_024305857.1	CDC7	8317	cell division cycle 7-related protein kinase isoform X1	[R].GMDSSTPKLTSDIQGHASHQPAISEKTDHK.[A]	S470; S471; T472; T476; S477; S484	1	1.157234977	0.842456828
XP_016878010.1	TJP1	7082	tight junction protein ZO-1 isoform X1	[R].HEEQPAPGYDTHGR.[L]	Y1258	1	4.356041637	0.085244168
NP_991403.1	LSR	51599	lipolysis-stimulated lipoprotein receptor isoform 2	[R].SGDLPYDGRLLEEAVR.[K]	Y586	1	#DIV/0!	#DIV/0!
NP_620709.1	MAPK9	5601	mitogen-activated protein kinase 9 isoform beta2	[R].TACTNFMMTPYVVTR.[Y]	Y/T	5	0.235282785	0.256185728
XP_005257569.1	GPRC5C	55890	G-protein coupled receptor family C group 5 member C isoform X1	[R].GVGYETILKEQK.[G]	Y424	2	#DIV/0!	#DIV/0!
NP_001302466.1	LDHB	3945	L-lactate dehydrogenase B chain isoform LDHBx	[K].MVVESAYEVIK.[L]	Y240	1	0.119102858	0.003223885
NP_000518.1	LDLR	3949	low-density lipoprotein receptor isoform 1 precursor	[K].TTEDEVHICHNQDGYSYPSR.[Q]	Y/S	10	1.318116501	0.833225426
XP_005263919.1	TBC1D8	11138	TBC1 domain family member 8 isoform X1	[K].LVVPWVDIQKLERTSNVFLTDTIRITTQNK.[E]		38	3.736436757	0.135617079
XP_011535388.1	MPP5	64398	MAGUK p55 subfamily member 5 isoform X1	[R].VYESIGQYGGETVK.[I]	Y243	4	0	0.004932076
NP_001074295.2	PRAG1	157285	inactive tyrosine-protein kinase PRAG1	[K].LNLSHSETNVHDESHFSYSLSPGNR.[H]	S/Y	2	0.323135775	0.321605972
NP_003019.2	SHB	6461	SH2 domain-containing adapter protein B	[K].GIQLYDTPYEPEGQSVDSDSESTVSPR.[L]	Y/T	4	#DIV/0!	#DIV/0!

Najar et al; Supplementary Table S1: List of phosphopeptides identified and quantified in LFQ based quantitative phosphoproteomic approach in gastric cancer cells (AGS) upon CAMKK2 inhibition.

XP_005266998.1	DCBLD1	285761	discoidin, CUB and LCCL domain-containing protein 1 isoform X1	[K].HSLSSGGFSPVAGVGAQDGDYQRPHSAQPADRGYDRPK.[A]	Y/S	3	#DIV/0!	#DIV/0!
NP_002341.1	LYN	4067	tyrosine-protein kinase Lyn isoform A	[R].TIYVRDPTSNK.[Q]	Y32	5	0	0.007698093
XP_011520133.1	STARD9	57519	stAR-related lipid transfer protein 9 isoform X1	[K].DSSEEFK.[L]	S1990	10	0.060534487	0.380700247
NP_001333375.1	AHNAK	79026	neuroblast differentiation-associated protein AHNAK isoform 1	[K].VKGEYDVTVPKLEGELK.[G]	T718	1	0	0.116200082
NP_001157789.1	FLNB	2317	filamin-B isoform 1	[R].SSTETCYSAIPK.[A]		8	0.309514468	0.011294859
XP_024304270.1	INPPL1	3636	phosphatidylinositol 3,4,5-trisphosphate 5-phosphatase 2 isoform X1	[R].LYEWISIDKDEAGAK.[S]	Y952	1	#DIV/0!	#DIV/0!
NP_001164185.1	BCAR1	9564	breast cancer anti-estrogen resistance protein 1 isoform 1	[R].RPGPGTLYDVPRER.[V]	Y433	1	#DIV/0!	#DIV/0!
