# Supplementary material for: Tyrosine Phosphorylation Profiling Revealed the Signaling Network Characteristics of CAMKK2 in Gastric Adenocarcinoma
Source: Front Genet. 2022 May 13;13:854764. doi: 10.3389/fgene.2022.854764 (PMC9136244; doi:10.3389/fgene.2022.854764)
Supplement: Supplementary file 5 [file Table2.PDF]

Najar et al; Supplementary Table S2: List of differentially phosphorylated peptides upon inhibition of CAMKK2 in gastric cancer cells (AGS).

Accession	Gene	Gene ID	Protein	Annotated Sequence	Modifications site	# PSMs	FC(T/C)	p-Value
NP_001341605.1	PRKCD	5580	protein kinase C delta type isoform a	[R].RSDSASSEPVGIYQGFEK.[K]	Y332	2	0	0.00001
NP_004422.2	EPHA2	1969	ephrin type-A receptor 2 isoform 1 precursor	[K].SEQLKPLKTYVDPHTYEDPNQAVLK.[F]	Y588	56	0.469	0.01886
NP_001158886.1	LDHA	3939	L-lactate dehydrogenase A chain isoform 3	[K].QVVESAYEVIK.[L]	Y268	2	0.363	0.04791
NP_000215.1	KRT18	3875	keratin, type I cytoskeletal 18	[R].STFSTNYR.[S]	Y13	26	0.529	0.03473
XP_011510318.1	PKP4	8502	plakophilin-4 isoform X1	[K].STTNYVDFYSTK.[R]	Y1168	40	0.645	0.04729
NP_001002857.1	ANXA2	302	annexin A2 isoform 2	[K].SYSPYDMLESIR.[K]	Y238	11	0.092	0.00089
NP_001078927.1	CTNND1	1500	catenin delta-1 isoform 1ABC	[K].SLDNNYSTPNER.[G]	Y904	20	0.169	0.00091
NP_001164563.1	CLDN2	9075	claudin-2	[R].SNYYDAYQAQPLATR.[S]	Y198	80	0.103	0.00126
XP_024306409.1	ERBB2	2064	receptor tyrosine-protein kinase erbB-2 isoform X1	[R].LLDIDETEYHADGGKVPIK.[W]	Y923	46	0.236	0.00938
XP_011510318.1	PKP4	8502	plakophilin-4 isoform X1	[R].NNYALNTTATYAEPYRPIQYR.[V]	Y470	9	0	0.00002
XP_011517854.2	KIAA1217	56243	sickle tail protein homolog isoform X1	[R].NEGFYADPYLYHEGR.[M]	Y467	16	0.431	0.03637
XP_011512733.1	ANKS1A	23294	ankyrin repeat and SAM domain-containing protein 1A isoform X1	[R].IHGSAAREEDEHPYELLLTAETK.[K]	Y476	1	0.170	0.04899
XP_016875215.1	PXN	5829	paxillin isoform X1	[R].VGEEEHVYSFPNK.[Q]	Y124	230	0.539	0.02261
NP_001291465.1	EPHA4	2043	ephrin type-A receptor 4 isoform a precursor	[R].VLEDDPEAAYTTR.[G]	T780	87	0.533	0.00897
NP_004422.2	EPHA2	1969	ephrin type-A receptor 2 isoform 1 precursor	[R].VLEDDPEATYTTSGGK.[I]	Y772	70	0.400	0.00811
NP_002341.1	LYN	4067	tyrosine-protein kinase Lyn isoform A	[R].VIEDNEYTAR.[E]	Y397	46	0.445	0.02007
NP_004422.2	EPHA2	1969	ephrin type-A receptor 2 isoform 1 precursor	[K].TYVDPHTYEDPNQAVLK.[F]	Y594	32	0.320	0.00112
NP_001339626.1	PTK2	5747	focal adhesion kinase 1 isoform e;ref|XP_024302967.1	[R].YMEDSTYYK.[A]	Y662	92	0.220	0.00022
NP_001191215.1	MUC1	4582	mucin-1 isoform 10 precursor	[R].YVPPSSTDRSPYEK.[V]	Y458	111	0.636	0.01810
NP_001339626.1	PTK2	5747	focal adhesion kinase 1 isoform e;ref|XP_024302967.1	[R].YMEDSTYYK.[A]	Y661	18	0.055	0.00048
NP_001339626.1	PTK2	5747	focal adhesion kinase 1 isoform e;ref|XP_024302967.1	[R].YMEDSTYYKASK.[G]	Y661,Y662	37	0.189	0.00386
XP_016882334.1	NUCB1	4924	nucleobindin-1 isoform X1	[R].YEMLKEHER.[R]	Y179	6	0.049	0.00317
XP_024306664.1	STAT3	6774	signal transducer and activator of transcription 3 isoform X5	[K].YCRPESQEHPEADPGSAAPYLK.[T]	Y737	137	0.348	0.00030
NP_001317366.1	PTPN11	5781	tyrosine-protein phosphatase non-receptor type 11 isoform 3	[R].VYENVGLMQQQK.[S]	Y584	9	0	0.04390
XP_005257673.2	STAT3	6774	signal transducer and activator of transcription 3 isoform X2	[K].YCRPESQEHPEADPGAAPYLK.[T]	Y705	76	0.296	0.01182
XP_024302967.1	PTK2	5747	focal adhesion kinase 1 isoform X1	[R].GSIDREDGSLQGPIGNQHIYQPVGKPDPAAPPK.[K]	Y925	45	0.291	0.02939
NP_005498.1	CFL1	1072	cofilin-1	[K].HELQANCYEEVKDR.[C]	Y140	6	0.096	0.01942
XP_024304270.1	INPPL1	3636	phosphatidylinositol 3,4,5-trisphosphate 5-phosphatase 2 isoform X1	[R].GLPSDYGRPLSFPPPR.[I]	Y1228	20	0.426	0.00217
XP_006721622.1	MAPK7	5598	mitogen-activated protein kinase 7 isoform X1	[R].GLCTSPAEHQYFMTEYVATR.[W]	T219	9	0.068	0.02911
XP_011512612.2	MAPK14	1432	mitogen-activated protein kinase 14 isoform X1	[R].HTDDEMTGYVATR.[W]	Y223	88	0.651	0.01761
XP_016865124.1	ERBIN	55914	erbin isoform X1	[R].AQIPEGDYLSYR.[E]	Y1104	25	0.356	0.00229
NP_001135865.1	NEDD9	4739	enhancer of filamentation 1 isoform 3	[R].DEAGLREKDYDFPPPMR.[Q]	Y241	80	0.309	0.03270
NP_003019.2	SHB	6461	SH2 domain-containing adapter protein B	[K].AGKGESAGYMEPYEAQR.[I]	Y268	44	0.375	0.01604
NP_001289958.1	PKP3	11187	plakophilin-3 isoform PKP3b	[R].ADYDTLSLR.[S]	Y191	14	0.367	0.01635

Najar et al; Supplementary Table S2: List of differentially phosphorylated peptides upon inhibition of CAMKK2 in gastric cancer cells (AGS).

NP_758869.1	CD46	4179	membrane cofactor protein isoform 2 precursor	[K].ADGGAEYATYQTK.[S]	Y387	93	0.413	0.01489
NP_001339626.1	PTK2	5747	focal adhesion kinase 1 isoform e	[R].FLIGNQHIYQPVGKPDPAAPPK.[K]	Y925	24	0.487	0.00626
NP_062565.2	PARD3	56288	partitioning defective 3 homolog isoform 1	[R].ERDYAEIQDFHR.[T]	Y1080	18	0.451	0.01276
XP_006721930.1	ITGB4	3691	integrin beta-4 isoform X3	[R].DYSTLTSVSSHDSR.[L]	Y1545	22	0.096	0.00372
NP_001078927.1	CTNND1	1500	catenin delta-1 isoform 1ABC	[R].HYEDGYPGGSDNYGSLSR.[V]	Y228	25	0.239	0.00151
XP_016865124.1	ERBIN	55914	erbin isoform X1	[R].RAQIPEGDYLSYR.[E]	Y1104	10	0.310	0.02205
NP_000692.2	ATP1A1	476	sodium/potassium-transporting ATPase subunit alpha-1 isoform a	[R].GIVVYTGDR.[T]	Y260	24	0.466	0.02797
XP_005257141.1	WIPF2	147179	WAS/WASL-interacting protein family member 2 isoform X1	[K].GSSGGYGSGGAALQPK.[G]	S70	21	0.447	0.02539
NP_001329.1	CXADR	1525	coxsackievirus and adenovirus receptor isoform 1 precursor	[K].TQYNQVPSEDFER.[T]	Y318	15	0.322	0.00699
NP_001333375.1	AHNAK	79026	neuroblast differentiation-associated protein AHNAK isoform 1	[K].VKGEYDVTVPKLEGELKGPK.[V]	Y715	8	0.306	0.02307
NP_001333375.1	AHNAK	79026	neuroblast differentiation-associated protein AHNAK isoform 1	[K].VKGEYDVTVPK.[L]	Y715	18	0.184	0.00105
XP_024308698.1	ITSN2	50618	intersectin-2 isoform X1	[K].REEPEALYAAVNK.[K]	Y968	8	0.118	0.00030
XP_005262892.1	FAT1	2195	protocadherin Fat 1 isoform X1	[K].KPLEEKPSQPYSAR.[E]	S4357	23	0.071	0.00331
XP_011518907.1	EPS8	2059	epidermal growth factor receptor kinase substrate 8 isoform X1	[R].HIDRNYEPLK.[T]	Y545	11	0.402	0.00597
XP_005251926.1	CEMIP2	23670	cell surface hyaluronidase isoform X1	[R].HPSGYVPGK.[V]	Y27	4	0.107	0.00250
XP_016875895.1	EFNB2	1948	ephrin-B2 isoform X1	[R].TADSVFCPHYEK.[V]	Y306	21	0.158	0.00255
XP_005257569.1	GPRC5C	55890	G-protein coupled receptor family C group 5 member C isoform X1	[R].GVGYETILK.[E]	Y424	11	0.527	0.04715
NP_001527.3	PRMT1	3276	protein arginine N-methyltransferase 1 isoform 1	[R].TGFSTSPESPYTHWK.[Q]	Y309	8	0.416	0.00633
NP_991403.1	LSR	51599	lipolysis-stimulated lipoprotein receptor isoform 2	[R].VLYYMEK.[E]	Y406	8	0.505	0.02139
XP_016881449.1	YES1	7525	tyrosine-protein kinase Yes isoform X1	[K].GAYSLSIR.[D]	Y194	20	0.369	0.03264
XP_011513778.1	TNS3	64759	tensin-3 isoform X2	[K].ESMCSTPAFPVSPETPYVK.[T]	Y971	6	0.154	0.00306
XP_024302967.1	PTK2	5747	focal adhesion kinase 1 isoform X1	[R].FLKPDVRLSRGSIDREDGSLQGPIGNQHIYQPVGKPDPAAPPK.[K]	Y925	15	0	0.00001
NP_000692.2	ATP1A1	476	sodium/potassium-transporting ATPase subunit alpha-1 isoform a	[R].KYGTDLSR.[G]	Y55	6	0.073	0.00141
NP_001171669.1	CTTN	2017	src substrate cortactin isoform c;ref| NP_005222.2	[K].NASTFEDVTQVSSAYQK.[T]	Y297	8	0.359	0.03418
NP_054772.1	FLVCR1	28982	feline leukemia virus subgroup C receptor-related protein 1	[K].GYLPLPR.[G]	Y22	14	0.299	0.00248
NP_001347.3	DDX3X	1654	ATP-dependent RNA helicase DDX3X isoform 1	[K].DKDAYSSFGSR.[SD]	Y69	7	0.175	0.03379
NP_006353.2	NXF1	10482	nuclear RNA export factor 1 isoform 1	[R].WKYGEGNRR.[S]	Y33	11	0.172	0.00077
XP_024305828.1	PEAK1	79834	pseudopodium-enriched atypical kinase 1 isoform X1	[R].STSSPYHAGNLLQR.[H]	Y880	8	0.171	0.02970
NP_001302466.1	LDHB	3945	L-lactate dehydrogenase B chain isoform LDHBx	[K].MVVESAYEVIK.[L]	Y240]	1	0.119	0.00322
XP_011535388.1	MPP5	64398	MAGUK p55 subfamily member 5 isoform X1	[R].VYESIGQYGGETVK.[I]	Y243	4	0	0.00493
NP_002341.1	LYN	4067	tyrosine-protein kinase Lyn isoform A	[R].TIYVRDPTSNK.[Q]	Y32	5	0	0.00770
NP_063937.2	GSK3A	2931	glycogen synthase kinase-3 alpha	[K].QLVRGEPNVSYICSR.[Y]	S278	198	15.997	0.01144
XP_016863455.1	GAB1	2549	GRB2-associated-binding protein 1 isoform X1	[K].SSGSGSSVADERVDYVVVDQQK.[T]	Y703	18	4.941	0.01656
XP_024305828.1	PEAK1	79834	pseudopodium-enriched atypical kinase 1 isoform X1	[K].SSAIRYQEVWTSSTSPR.[Q]	Y531	23	4.641	0.00521
XP_024302814.1	LCK	3932	tyrosine-protein kinase Lck isoform X1	[R].NLDNGGFYISPR.[I]	Y250	30	1.582	0.01523
NP_001292553.1	CALM2	805	calmodulin-2 isoform 1	[R].VFDKDGNGYISAAELR.[H]	Y148	43	2.559	0.00885
NP_004094.3	PTK2B	2185	protein-tyrosine kinase 2-beta isoform a	[R].YIEDEDYYKASVTRLPIK.[W]	Y579	16	4.612	0.04029
NP_004330.1	PTTG1IP	754	pituitary tumor-transforming gene 1 protein-interacting protein isoform 1 precursor	[K].YGLFKEENPYAR.[F]	Y174	73	2.137	0.00922

Najar et al; Supplementary Table S2: List of differentially phosphorylated peptides upon inhibition of CAMKK2 in gastric cancer cells (AGS).

XP_024305828.1	PEAK1	79834	pseudopodium-enriched atypical kinase 1 isoform X1	[R].YQEVWTSSTSPR.[Q]	Y531	32	2.166	0.01691
XP_011513536.1	COBL	23242	protein cordon-bleu isoform X1	[R].TSSQYVASAIAK.[R]	Y964]	9	1.675	0.04943
XP_016878010.1	TJP1	7082	tight junction protein ZO-1 isoform X1	[R].FTPKPYTSSARPFER.[K]	Y1642	15	2.553	0.04119
NP_003080.2	SNRNP70	6625	U1 small nuclear ribonucleoprotein 70 kDa isoform 1	[R].EFEVYGPIKR.[I]	Y126	74	1.536	0.01943
NP_002957.1	S100A10	6281	protein S100-A10	[K].FAGDKGYLTKEDLR.[V]	Y25	30	1.590	0.01698
NP_001307847.1	CDK1	983	cyclin-dependent kinase 1 isoform 1	[K].IGEGTYGVVYKGR.[H]	T14	111	9.216	0.03533
NP_001307847.1	CDK1	983	cyclin-dependent kinase 1 isoform 1	[K].IEKIGEGTYGVVYK.[G]	T14	60	7.746	0.03704
NP_001307847.1	CDK1	983	cyclin-dependent kinase 1 isoform 1	[K].IEKIGEGTYGVVYK.[G]	T14	94	5.560	0.01383
NP_002737.2	MAPK3	5595	mitogen-activated protein kinase 3 isoform 1		T202	2	12.753	0.02698
